# Functional Analysis of the *Aspergillus nidulans* Kinome

**DOI:** 10.1371/journal.pone.0058008

**Published:** 2013-03-07

**Authors:** Colin P. De Souza, Shahr B. Hashmi, Aysha H. Osmani, Peter Andrews, Carol S. Ringelberg, Jay C. Dunlap, Stephen A. Osmani

**Affiliations:** 1 Department of Molecular Genetics, The Ohio State University, Columbus, Ohio, United States of America; 2 Department of Genetics, Geisel School of Medicine at Dartmouth, Hanover, New Hampshire, United States of America; 3 Institute for Quantitative Biomedical Sciences, Geisel School of Medicine at Dartmouth, Hanover, New Hampshire, United States of America; University of Wisconsin - Madison, United States of America

## Abstract

The filamentous fungi are an ecologically important group of organisms which also have important industrial applications but devastating effects as pathogens and agents of food spoilage. Protein kinases have been implicated in the regulation of virtually all biological processes but how they regulate filamentous fungal specific processes is not understood. The filamentous fungus *Aspergillus nidulans* has long been utilized as a powerful molecular genetic system and recent technical advances have made systematic approaches to study large gene sets possible. To enhance *A. nidulans* functional genomics we have created gene deletion constructs for 9851 genes representing 93.3% of the encoding genome. To illustrate the utility of these constructs, and advance the understanding of fungal kinases, we have systematically generated deletion strains for 128 *A. nidulans* kinases including expanded groups of 15 histidine kinases, 7 SRPK (serine-arginine protein kinases) kinases and an interesting group of 11 filamentous fungal specific kinases. We defined the terminal phenotype of 23 of the 25 essential kinases by heterokaryon rescue and identified phenotypes for 43 of the 103 non-essential kinases. Uncovered phenotypes ranged from almost no growth for a small number of essential kinases implicated in processes such as ribosomal biosynthesis, to conditional defects in response to cellular stresses. The data provide experimental evidence that previously uncharacterized kinases function in the septation initiation network, the cell wall integrity and the morphogenesis Orb6 kinase signaling pathways, as well as in pathways regulating vesicular trafficking, sexual development and secondary metabolism. Finally, we identify ChkC as a third effector kinase functioning in the cellular response to genotoxic stress. The identification of many previously unknown functions for kinases through the functional analysis of the *A. nidulans* kinome illustrates the utility of the *A. nidulans* gene deletion constructs.

## Introduction

The filamentous fungi have critical ecological roles both as plant symbionts and recyclers of biomass. They have additional economic impact via their beneficial industrial applications and via their detrimental effects as pathogens and agents of food spoilage [1]. The filamentous ascomycete *Aspergillus nidulans* has historically been a productive model system for the discovery of genes involved in fungal specific processes such as secondary metabolite production as well as universal regulators of the cell cycle and cytoskeleton [2]–[4]. Along with a growing number of Aspergilli and other filamentous fungi, the *A. nidulans* genome has been sequenced and the extensive annotation of this organism is being further refined by recently available RNA-Seq data [5]–[11]. However, despite these advances, the majority of filamentous fungal genes still await characterization. To provide tools to enhance this effort we describe the generation of gene knock-out constructs for 93.3% of the 10,560 predicted *A. nidulans* genes. To demonstrate the utility of these constructs and to better understand fungal kinase biology, we have created and phenotypically characterized gene deletion strains for 128 *A. nidulans* protein kinases.

Reversible protein phosphorylation plays a critical role in the regulation of virtually all eukaryotic biological processes [12]–[18]. Reflecting this, a significant proportion of eukaryotic genomes encode enzymes regulating phosphorylation. For example, the 131 predicted protein kinases and 28 phosphatase catalytic subunits respectively comprise 1.25% and 0.27% of the *A. nidulans* genome [19]. Based on their catalytic domains conventional protein kinases can be classified into the following groups; AGC (protein kinase A, G or C), CAMK (Ca2+/calmodulin-dependent protein kinases), CK1 (casein kinase 1), CMGC (cyclin-dependent, mitogen-activated, glycogen synthase and cyclin-dependent protein kinase-like kinases), STE (sterile kinases), RGC (receptor guanylate cyclase kinases), TK (tyrosine kinases), TKL (Tyrosine like kinases), and others [20]. Additional atypical kinases, which show little or no similarity to the above conventional kinases, include the PIKK (Phosphatidylinositol kinase-related kinases), PDHK (pyruvate dehydrogenase kinases), RIO (right open reading frame) and histidine kinases [20]. Interestingly, recent analysis indicates that filamentous fungi contain novel families of kinases not related to the above families of kinases [9]–[11], [14], [16].

Cells respond to environmental stimuli through signaling pathways often involving kinase cascades which transmit external cellular signals to the nucleus [9], [21]. Examples of this include the MAPK (mitogen activated protein kinases) signaling pathways which are found throughout eukaryotes [22], [23]. Although absent from mammals, many eukaryotes also utilize two component cell signaling systems consisting of a histidine kinase and a response regulator [24], [25]. Relative to yeast, filamentous fungi encode an expansion of histidine kinases which are of considerable interest as anti-fungal targets [23], [26]. Unlike other protein kinases which phosphorylate serine, threonine and/or tyrosine residues, histidine kinases transfer phosphate groups between specific histidine and aspartate residues [24], [25]. In addition, individual cell signaling pathways can communicate with each other thereby building a complex network to ultimately control gene expression and other cellular functions.

Cell growth and the cell cycle are also in large part coordinated by kinases. For example, the Cdk1 mitotic kinase is kept inactive during G2 by inhibitory phosphorylation carried out the Wee1 kinase. Removal of Cdk1 inhibitory phosphorylation by the Cdc25 phosphatase activates Cdk1 thereby promoting entry into mitosis [3], [27]. In *A. nidulans, Magnaporthe grisea* and likely other filamentous fungi, activation of the NIMA kinase is additionally required to promote mitotic entry [3], [28], [29]. Kinases also play central roles following exposure of cells to genotoxic or osmotic stress [30], [31]. For example, when DNA is damaged, the ATM and/or ATR PIKK kinases phosphorylate the downstream effector kinases Chk1 and Chk2 [31]. These effector kinases then signal to the DNA repair and cell cycle machinery to arrest the cell cycle until DNA repair is completed.

Several kinases have been intensively studied in filamentous fungi including *Neurospora crassa* Cot-1 and *A. nidulans* NIMA which are the founding members of their respective kinase families [32], [33]. However, despite their importance, only 44 of the 131 kinases encoded by the *A. nidulans* kinome have been genetically characterized. By phylogenetic analysis we show that this kinome encodes 11 predicted filamentous fungal kinases (Ffks) not found outside the filamentous fungi. Using deletion constructs we describe the generation and phenotypic analysis of mutants for 128 *A. nidulans* kinases. This analysis defined 25 kinases with an essential function and a further 43 kinases whose deletion lead to at least one phenotype. We have expanded on recent studies of the kinomes of *N. crassa* [16] and the wheat scab fungus *Fusarium graminearum* [14] by including the PIKK and histidine kinases, and by utilizing heterokaryon rescue to phenotypically analyze cells lacking the essential *A. nidulans* kinases. Our phenotypic analysis has implicated *A. nidulans* kinases in regulating ribosomal biogenesis, mRNA splicing and the unfolded protein response, and revealed previously unknown roles for kinases in the regulation of vegetative growth, septation, polarized growth, the cell cycle, development, secondary metabolite production, the DNA damage response, and the cellular response to osmotic stress.

## Results

### Global Production of *A. nidulans* Gene Deletion Constructs

**Figure 1:**
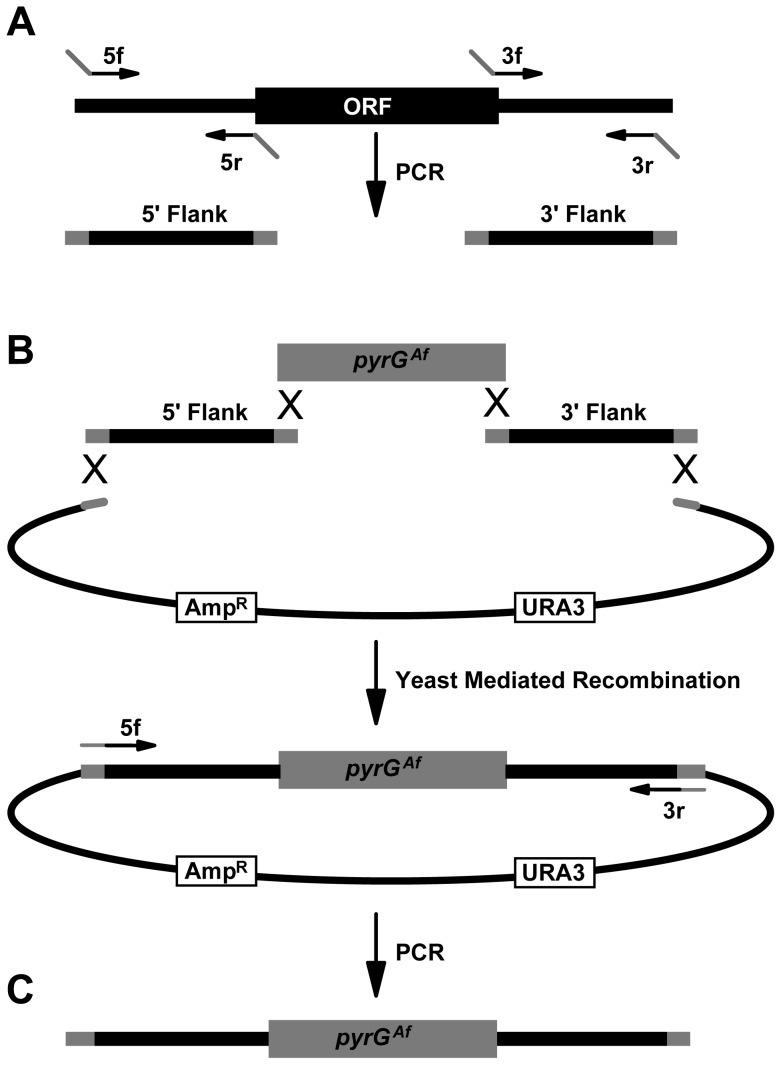
Strategy for generation of deletion constructs. (A) Amplification of 5′ and 3′ gene specific flanking fragments from *A. nidulans* genomic DNA. Primers 5r and 3f have 5′ extensions complementary to the *pyrG* ^Af^ cassette, whereas 5f and 3r have 5′ extensions complementary to the vector. (B) Co-transformation of yeast with the 5′ flank and 3′ flank together with the *pyrG* ^Af^ cassette and a gapped yeast shuttle vector to generate a plasmid containing the gene specific deletion construct [34]. (C) The final linear deletion construct is generated by PCR from the yeast DNA with primers 5f and 3r.

To generate gene deletion constructs for all *A. nidulans* genes we utilized a high throughput platform used to generate similar constructs for *Neurospora crassa* [34]. Constructs were designed to replace the open reading frame (ORF) of each *A. nidulans* gene with *pyrG^Af^* (*A. fumigatus* orotidine 5′-phosphate carboxylase which complements the *A. nidulans pyrG*89 or Δ*pyrG* mutations) (Figure 1). The *pyrG* nutritional marker was chosen as it provides a robust means to identify and phenotypically study gene deletions which cause lethality using the heterokaryon rescue technique [35], and additionally allows counter selection using 5-FOA (5-Fluoroorotic Acid) [36]. Deletion constructs consisted of 0.6–1 kb of 5′ sequence flanking the target gene ORF (5′ flank), *pyrG^Af^*, and 0.6–1 kb of 3′ sequence flanking the target gene ORF (3′ flank), and were generated by PCR and yeast recombinational cloning (Figure 1). For each deletion construct, four primers, designated 5f, 5r, 3f and 3r, were designed as described using some modifications [34] (materials and methods). Extensions on the 5r and 3r primers complementary to the *pyrG^Af^* cassette, and on the 5f and 3r primers complementary to a yeast vector, facilitated yeast recombinational cloning (Figure 1). Primer design was successful for 95.4% of the 10,560 ORFs predicted in the Broad *A. nidulans* database (version 2) and these primers are listed in Table S1 (http://www.fgsc.net/Aspergillus/KO_Cassettes.htm). Design failures were attributable to the close proximity of genes to contig ends or because the sequence flanking the gene was not compatible with the stringent parameters of the design algorithm. The final linear deletion constructs were amplified by PCR with a 95.3% success rate. Of the 263 genes for which yeast recombinational cloning failed, 249 were successfully generated by fusion PCR [37], [38] using the same primers. In total, full length deletion constructs were generated for 9,851 *A. nidulans* genes (93.3%) and are available under request from the Fungal Genetics Stock Center (http://www.fgsc.net/Aspergillus/KO_Cassettes.htm).

### The Aspergillus Nidulans Kinome

**Figure 2:**
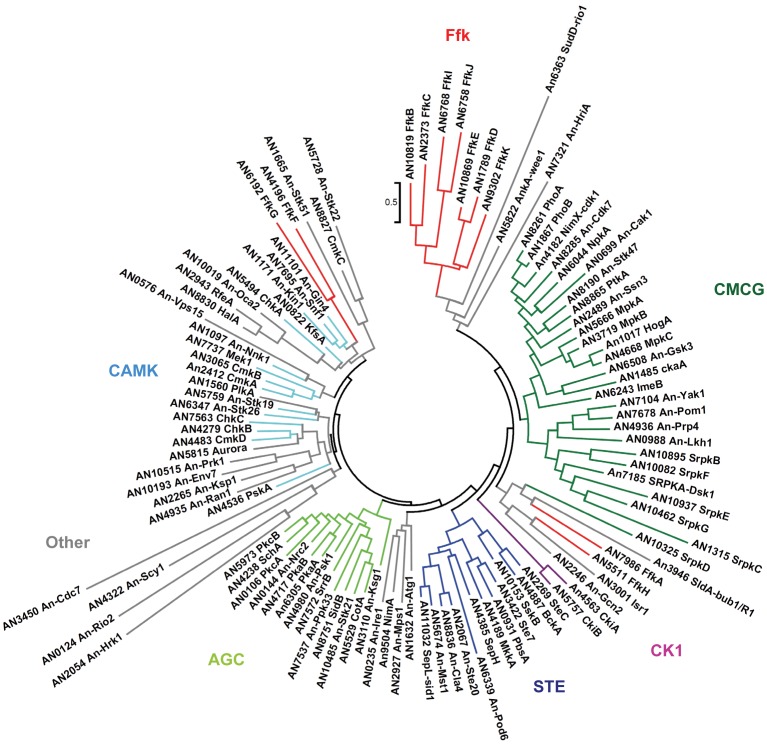
Phylogenetic analysis of the *A. nidulans* protein kinases. Kinase domain alignment generated using ClustalW (http://www.phylogeny.fr) and tree visualization using MEGA version 5 maximum likelihood analyses. Domains of the atypical PIKK, PDHK and histidine kinases were omitted. The major families of kinases are indicated in different colors. The Ffk kinases are indicated in red. Note that FfkB, FfkC, FfkD, FfkE, FfkI, FfkJ and FfkK are more similar to each other than to other kinases.

Although protein kinases are involved in the regulation of essentially all biological processes, many kinases remain poorly studied. The *A. nidulans* genome encodes 131 predicted protein kinases, of which only 44 (33.6%) have been genetically characterized. Analysis of the catalytic domains of these kinases by BLAST comparison with the Salk Institute’s kinome database (http://kinase.com/ [20]) allowed 120 to be classified into the following groups of kinases: 13 AGC (protein kinase A, G or C), 15 CAMK (Ca2+/calmodulin-dependent protein kinases), 2 CK1 (casein kinase 1), 27 CMGC (cyclin-dependent, mitogen-activated, glycogen synthase and cyclin-dependent protein kinase-like kinases), 12 STE (sterile kinases), 11 atypical, 15 atypical histidine kinases and 25 other (Table 1, Figure 2). We assigned names for previously unnamed kinases based on the most well characterized orthologous kinase present in other organisms (* in Table 1).

**Table 1:** Summary of *A. nidulans* protein kinase classification and phenotypes.

Systematic name	*A. nidulans* name	*S. cerevisiae* orthologue	*S. pombe* orthologue		*N. crassa* orthologue	Group/Family/Sub-Family			Deletion		Phenotype	
AN4238	SchA	SCH9	sck1		NCU03200 stk-10	AGC/Akt			Viable			
AN5973	PkcB	YPK1 (BH)	gad8		NCU07280 stk-50	AGC/Akt			Lethal		Microcolony	
AN5529	CotA	CBK1 (BH)	orb6		NCU07296 cot-1	AGC/NDR			Lethal		Microcolony; brown; Polarity defect	
AN10485	An-Stk21*	CBK1 (BH)	orb6 (BH)		NCU03242 stk-21	AGC/NDR			Viable			
AN7572	SrrB	RIM15	ppk18		NCU07378 stk-12	AGC/NDR			Viable		NaCl^s^	
AN8751	SidB	DBF20/2	sid2		NCU09071 dbf-2	AGC/NDR			Viable		Strong growth defect-septation and conidiation; NaCl^s^	
AN3110	An-Ksg1*	PKH1/2 (BH)	ksg1		NCU03571 stk-23	AGC/PDK1			Lethal		Microcolony	
AN4717	PkaB	TPK1 (BH)	pka1 (BH)		NCU00682 pkac-2	AGC/PKA			Viable			
AN6305	PkaA	TPK2	pka1		NCU06240 pkac-1	AGC/PKA			Viable		Strong growth defect; Suc^Rem^; NaCl^Rem^	
AN0106	PkcA	PKC1 (BH)	pck2		NCU06544 pkc	AGC/PKC/PKC			Lethal		Swollen	
AN4980	An-Psk1*	YPK3 (BH)	psk1		NCU03197 stk-11	AGC/RSK			Viable			
AN0144	An-Nrc2*	FPK1/KIN82 (BH)	ppk14		NCU01797 nrc-2	AGC/RSK			Viable		Moderate growth defect	
AN7537	An-Ppk33*	YPK2 (BH)	ppk33		NCU07062 stk-49	AGC/YANK			Viable		NaCl^s^	
AN2412	CmkA	CMK1/2	cmk1 (BH)		NCU09123 camk-1	CAMK/CAMK1			Viable		Moderate growth defect; Increased pigment production	
AN3065	CmkB	CMK1/2 (BH)	cmk1		NCU02283 camk-2	CAMK/CAMK1			Viable			
AN4483	CmkD*	RCK1/2 (BH)	srk1		NCU09212 camk-4	CAMK/CAMK1			Viable			
AN7695	An-Snf1*	SNF1	ssp2		NCU04566 prk-10	CAMK/CAMKL/AMPK			Viable			
AN5494	ChkA	CHK1	chk1		NCU08346 mus-58	CAMK/CAMKL/CHK1			Viable		HU; DEO	
AN11101	An-Gin4*	GIN4	cdr2 (BH)		NCU09064 stk-53	CAMK/CAMKL/GIN4			Viable		Early sexual development	
AN1171	An-Kin1*	KIN1 (BH)	kin1		NCU04747 stk-31	CAMK/CAMKL/Kin1			Viable		Moderate growth defect	
AN0822	KfsA	KIN4 (BH)	ppk1 (BH)		NCU00914 stk-16	CAMK/CAMKL/Kin4			Viable		Moderate growth defect	
AN5759	An-Stk19*	YPL150W (BH)	ppk16 (BH)		NCU02245 stk-19	CAMK/CAMKL/MARK			Viable			
AN4536	An-Psk1*	PSK1/2	ppk6		NCU06249 stk-40	CAMK/CAMKL/PASK			Viable			
AN6347	An-Stk26*	PRR1 (BH)	ppk27 (BH)		NCU04143 stk-26	CAMK/CAMK-Unique			Viable			
AN7737	An-Mek1*	MEK1	mek1		NCU06486 stk-43 (BH)	CAMK/CAMK-Unique			Viable			
AN1097	An-Nnk1*	NNK1	kin1 (BH)		None	CAMK/CAMK-Unique			Viable			
AN4279	ChkB	DUN1	cds1		NCU02814 prd-4	CAMK/RAD53			Viable		HU	
AN7563	ChkC*	RAD53 (BH)	cds1 (BH)		NCU02751 mus-59	CAMK/RAD53			Viable		HU	
AN4563	CkiA	HRR25 (BH)	hhp1		NCU00685 ck-1a	CK1/CK1/CK1-D			Lethal		Short germling arrest	
AN5757	CkiB	YCK2	cki1		NCU04005 ck-1b	CK1/CK1/CK1-G			Viable		Strong growth defect	
AN0699	An-Cak1*	CDC28 (BH)	csk1		NCU04426 div-4	CMGC/CDK/CDC2			Viable		Strong growth defect; Het	
AN4182	NimX^Cdk1^	CDC28	cdc2		NCU09778 cdc28	CMGC/CDK/CDC2			Lethal		Cell cycle	
AN1867	PhoB	PHO85	pef1		NCU07580 mdk-1	CMGC/CDK/CDK5			Viable			
AN8261	PhoA	PHO85	pef1		NCU07580 mdk-1	CMGC/CDK/CDK5			Viable		NaCl^s^	
AN8285	An-Cdk7*	KIN28 (BH)	mcs6		NCU03659 prk-3	CMGC/CDK/CDK7			Lethal		Swollen, Enlarged vacuoles	
AN2489	An-Ssn3*	SSN3	srb10		NCU07172 stk-8	CMGC/CDK/CDK8			Viable		Moderate growth defect	
AN8865	PtkA	SGV1 (BH)	cdk9		NCU01435 stk-1	CMGC/CDK			Viable		Strong growth defect; NaCl^s^	
AN8190	An-Stk47*	CTK1 (BH)	lsk1 (BH)		NCU06685 stk-47	CMGC/CDK/CRK7			Viable		Strong growth defect; NaCl^s^	
AN6044	NpkA	CTK1 (BH)	ppk23		NCU07880 prk-6	CMGC/CDK			Viable			
AN1485	An-Cka1 *	CKA1/2 (BH)	cka1		NCU03124 cka	CMGC/CK2			Lethal		Microcolony	
AN0988	An-Lkh1*	KNS1 (BH)	lkh1		NCU00230 prk-4	CMGC/CLK			Viable		Strong growth defect	
AN4936	An-Prp4*	YAK1 (BH)	prp4		NCU10853 stk-57	CMGC/DYRK/PRP4			Lethal		Short germling arrest; Swollen	
AN7104	An-Yak1*	YAK1	ppk15 (BH)		NCU07872 prk-2	CMGC/DYRK/YAK			Viable		Moderate growth defect; Yellow	
AN7678	An-Pom1*	YAK1 (BH)	pom1 (BH)		NCU06638 stk-46	CMGC/DYRK/YAK			Viable			
AN6508	An-Gsk3*	RIM11 (BH)	gsk3		NCU04185 gsk-3	CMGC/GSK			Viable		Strong growth defect; brown; Conidiation defect; Suc^Rem^	
AN1017	HogA	HOG1	sty1		NCU07024 os-2	CMGC/MAPK/ERK			Viable		NaCl^s^	
AN3719	MpkB	FUS3 (BH)	spk1		NCU02393 mak-2	CMGC/MAPK/ERK			Viable		Moderate growth defect; Arrested sexual development	
AN4668	MpkC	HOG1 (BH)	sty1 (BH)		NCU07024 os-2	CMGC/MAPK/ERK			Viable			
AN5666	MpkA	SLT2 (BH)	pmk1		NCU11376 UN	CMGC/MAPK/ERK			Viable		Branched germling arrest; NaCl^rem^; Suc^rem^	
AN6243	ImeB	IME2 (BH)	pit1 (BH)		NCU01498 ime-2	CMGC/RCK/MAK			Viable		Moderate growth defect	
AN7185	SrpkA^Dsk1^*	SKY1	dsk1		NCU09202 mdk-2	CMGC/SRPK			Viable		Moderate growth defect; NaCl^s^	
AN10895	SrpkB*	SKY1 (BH)	lkh1 (BH)		NCU09202 mdk-2 (BH)	CMGC/SRPK			Viable			
AN1315	SrpkC*	SKY1 (BH)	dsk1 (BH)		NCU09202 mdk-2 (BH)	CMGC/SRPK			Viable			
AN10325	SrpkD*	SKY1 (BH)	dsk1 (BH)		NCU05658 stk-36 (BH)	CMGC/SRPK			Viable			
AN10937	SrpkE*	SKY1 (BH)	dsk1 (BH)		NCU09202 mdk-2 (BH)	CMGC/SRPK			Viable			
AN10082	SrpkF*	SKY1 (BH)	dsk1 (BH)		NCU09202 mdk-2 (BH)	CMGC/SRPK			Viable			
AN10462	SrpkG*	SKY1 (BH)	dsk1 (BH)		NCU09202 mdk-2 (BH)	CMGC/SRPK			Viable			
AN5815	An-Aurora*	IPL1	ark1		NCU00108 stk-13	Other/Aur			Lethal		Nulls not generated from heterokaryons	
AN3946	SldA	BUB1 (BH)	bub1		NCU10043 UN	Other/BUB			Viable		Moderate growth defect; Ben; NaCl^s^; Colony color	
AN5728	An-Stk22*	SAK1 (BH)	ssp1 (BH)		NCU03523 stk-22	Other/CAMKK/ELM			Viable			
AN8827	CmkC	TOS3 (BH)	ppk34 (BH)		NCU06177 camk-3	Other/CAMKK/ELM			Viable		NaCl^s^	
AN3450	An-Cdc7*	CDC7 (BH)	hsk1		NCU11410 cdc7	Other/CDC7			Lethal		Cell cycle; Swollen	
AN10019	An-Oca2*	HRK1	oca2		NCU04335 stk-30	Other/HAL			Viable			
AN2943	RfeA	SAT4 (BH)	hal4 (BH)		NCU06179 stk-5 (BH)	Other/HAL			Viable			
AN8830	HalA	SAT4 (BH)	hal4		NCU06179 stk-5	Other/HAL			Viable		Moderate growth defect; NaCl^s^	
AN2054	An-Haspin*	Alk1/2	hrk1		NCU00407 UN	Other/Haspin			Viable			
AN1665	An-Stk51*	IKS1 (BH)	ssp1 (BH)		NCU08177 stk-51	Other/IKS			Viable			
AN0235	IreA*	IRE1	ire1		NCU02202 stk-14	Other/IRE			Lethal		Swollen	
AN10515	An-Prk1*	PRK1/ARK1	ppk30 (BH)		NCU06202 stk-38	Other/NAK/NAK			Viable		Strong growth defect	
AN10193	An-Env7*	ENV7	ppk13		NCU07399 stk-9	Other/NAK			Viable			
AN9504	NimA	KIN3 (BH)	fin1		NCU3187 nim-1	Other/NEK/NEK2			Lethal		Cell cycle	
AN2246	An-Gcn2*	GCN2	gcn2		NCU01187 cpc-3	Other/PEK/GCN2			Viable			
AN7321	An-HriA*	GCN2 (BH)	hri1/2		NCU01187 (BH)	Other/PEK/HRI			Viable			
AN1560	PlkA	CDC5 (BH)	plo1		NCU09258 cdc5	Other/PLK			Viable		Strong growth defect; Early sexual development; NaCl^s^	
AN2265	An-Ksp1*	KSP1 (BH)	ksp1		NCU06230 stk-39	Other/RAN			Viable			
AN4935	An-Ran1*	VHS1/SKS1	ran1	NCU04990 stk-17/fi			Other/RAN	Viable				
AN4322	An-Scy1*	SCY1	ppk32	NCU04755 stk-32			Other/SCY1	Viable				
AN2927	An-Mps1*	MPS1 (BH)	mph1 (BH)	NCU00978 UN			Other/TTK	Lethal		Nulls not generated from heterokaryons		
AN1632	An-Atg1*	ATG1	atg1	NCU00188 apg-1			Other/ULK/ULK	Viable				
AN0576	An-Vps15*	VPS15	ppk19	NCU06626 stk-45			Other/VPS15	Lethal		Microcolony		
AN3001	An-Isr1*	Isr1 (BH)	None	None			Other/Other-Unique	Viable				
AN5822	An-Wee1	SWE1 (BH)	wee1	NCU04326 stk-19			Other/WEE	Lethal		Cell cycle		
AN4385	SepH	CDC15	cdc7	NCU01335 cdc15			STE/STE11/CDC15	Lethal		Strong growth defect-septation and conidiation; NaCl^s^; Het		
AN10153	SskB	SSK2 (BH)	wis4	NCU03071 os-4			STE/STE11/STE11	Viable		NaCl^s^; Suc^s^		
AN2269	SteC	STE11	byr2	NCU06182 nrc-1			STE/STE11	Viable		Moderate growth defect; Arrested sexual development		
AN4887	BckA	BCK1 (BH)	mkh1 (BH)	NCU02234 mik-1			STE/STE11	Lethal		Branched germling arrest; NaCl^rem^; Suc^rem^		
AN6339	An-Pod6*	KIC1 (BH)	nak1 (BH)	NCU11235 pod-6			STE/STE20	Lethal		Microcolony; brown; Polarity defect		
AN2067	An-Ste20*	STE20 (BH)	shk1	NCU03894 stk-4			STE/STE20/PAKA	Viable		Increased pigment production		
AN8836	An-Cla4*	CLA4	shk2	NCU00406 vel			STE/STE20/PAKA	Viable		Strong growth defect		
AN11032	SepL^sid1^	SPS1 (BH)	sid1	NCU04096 prk-9			STE/STE20/YSK	Viable		Strong growth defect-septation and conidiation; NaCl^s^		
AN5674	An-Mst1	KIC1 (BH)	ppk11 (BH)	NCU00772 mst-1			STE/STE20/YSK	Viable		Early but incomplete sexual development		
AN0931	PbsA	PBS2	wis1	NCU00587 os-5			STE/STE7/STE7	Viable		NaCl^s^; Suc^s^		
AN3422	Ste7	STE7	byr1	NCU04612 mek-2			STE/STE7	Viable		Moderate growth defect; Arrested sexual development		
AN4189	MkkA	MKK1	pek1	NCU06419 mek-1			STE/STE7	Lethal		Branched germling arrest; NaCl^rem^; Suc^rem^		
AN7986	FfkA*	None	None	None			Unclassified; Ank	Viable				
AN10819	FfkB*	None	None	NCU06582 (BH)			Unclassified; Ank	Viable				
AN2373	FfkC*	None	None	NCU06583 (BH)			Unclassified; Ank	Viable				
AN1789	FfkD*	None	None	NCU11331 (BH) UN			Unclassified	Viable				
AN10869	FfkE*	None	None	NCU11331 (BH) UN			Unclassified	Viable				
AN4196	FfkF*	None	None	NCU03250 UN			Unclassified	Viable				
AN6192	FfkG*	None	None	NCU05260 UN			Unclassified	Viable				
AN5511	FfkH*	None	None	NCU05638 stk-34 (BH)			Unclassified	Viable				
AN6768	FfkI*	None	None	None			Unclassified	Viable				
AN6758	FfkJ*	None	None	None			Unclassified	ND				
AN9302	FfkK*	None	None	None			Unclassified	ND				
AN10800	PkpA*	PKP1	Pkp1 (BH)	NCU11744 stk-58			Atypical/PDHK	Viable				
AN9461	PkpB*	PKP2	Pkp1 (BH)	NCU03796 stk-24			Atypical/PDHK	ND				
AN6207	PkpC*	PKP2 (BH)	Pkp1	NCU06760 stk-48			Atypical/PDHK	Viable				
AN4278	An-Stt4*	STT4	stt4	NCU09367 UN			Atypical/PIKK	Lethal^ k^		Micro; V; Cell cycle		
AN4709	An-Vps34	VPS34	pik3	NCU00656 UN			Atypical/PIKK	Lethal		Microcolony		
AN10791	An-Lsb6*	LSB6	lsb6	NCU04355 UN			Atypical/PIKK	Viable		Strong growth defect; Het		
AN0038	AtmA	TEL1	tel1	NCU00274 mus-21			Atypical/PIKK/ATM	Viable		Camp		
AN6975	UvsB	MEC1	rad3	NCU11188 mus-9			Atypical/PIKK/ATR	Viable		Moderate growth defect; HU; DEO; Camp; Colony color		
AN5982	TorA	TOR1/2	tor1/2	NCU05608 div-18			Atypical/PIKK/FRAP	Lethal		Short germling arrest; Early septation		
AN6363	SudD	RIO1(BH)	SPAC10F6.10	NCU 08767 stk-52			Atypical/RIO/RIO1	Lethal		Short germling arrest; Swollen		
AN0124	An-Rio2*	RIO2	SPBC1703.05	NCU07722 rgb-40			Atypical/RIO/RIO2	Lethal		Short germling arrest		
AN5296	TcsA	SKN7 (BH)	mak2 (BH)	NCU07221			Atypical/HisK	Viable				
AN1800	TcsB	SLN1	mak2 (BH)	NCU04615			Atypical/HisK	Viable				
AN7945	hk-2	SNL1 (BH)	mak2 (BH)	NCU00939 dcc1			Atypical/HisK	Viable				
AN2581	hk-8-1	SSK1 (BH)	mcs4 (BH)	NCU09520 (BH)			Atypical/HisK	Viable				
AN4113	hk-8-2	SSK1 (BH)	mcs4 (BH)	NCU09520 (BH)			Atypical/HisK	Viable				
AN6820	hk-8-3	SSK1 (BH)	mcs4 (BH)	NCU09520 (BH)			Atypical/HisK	Viable				
AN4818	hk-8-4	SSK1 (BH)	mak2 (BH)	NCU09520 (BH)			Atypical/HisK	Viable				
AN3214	hk-8-5	SKN7 (BH)	mak2 (BH)	NCU09520 (BH)			Atypical/HisK	Viable				
AN2363	hk-8-6	SNL1 (BH)	mak2 (BH)	NCU09520 (BH)			Atypical/HisK	Viable				
AN9048	hk-8-7	SNL1 (BH)	mak1 (BH)	NCU09520 (BH)			Atypical/HisK	Viable				
AN4447	hk-9*	SNL1 (BH)	mak1 (BH)	NCU09520 (BH)			Atypical/HisK	Viable				
AN4479	NikA	SKN7 (BH)	mak2 (BH)	NCU02815 nik1			Atypical/HisK	Viable		Strong growth defect		
AN3102	PhkA	SNL1 (BH)	mak3	NCU01823			Atypical/HisK	Viable ^k^				
AN3101	PhkB	SSK1 (BH)	mak1	NCU01833 nik2			Atypical/HisK	Viable				
AN9008	FphA	SNL1 (BH)	mak2 (BH)	NCU04834 phy1			Atypical/HisK	Viable				

**Group abbreviations**: AGC (protein kinase A, G or C); CK1 (casein kinase 1); CAMK (Ca2+/calmodulin-dependent protein kinase); CMGC (cyclin-dependent, mitogen-activated, glycogen synthase and cyclin-dependent protein kinase-like kinases); STE (sterile kinases); HisK (histidine kinase). **Family abbreviations**: Akt (oncogene protein of v-akt); NDR (nuclear Dbf2 related); PDK1(phosphoinoside-dependent protein kinase); PKA (protein kinase A); PKC (protein kinase C); RSK (ribosomal S6 kinase); YANK (yet another novel kinase); CAMKL (CAMK-like kinase); CK (casein kinase); CDK (cyclin-dependent protein kinase); CLK (CDK-like kinase); DYRK, (dual-specificity tyrosine phosphorylation-regulated kinase); GSK (glycogen synthase kinase); MAPK (mitogen-activated protein kinase); RCK (related to murine RCK); SRPK (serine-arginine protein kinase); AUR (Aurora); CAMKK (CAMK kinase); CDC (cell division cycle); HAL (halotolerance); IRE (inositol-requiring protein); NAK (NF-κB-activating kinase); NEK (NIMA-related kinase); PEK (pancreatic alpha-subunit of eukaryotic initiation factor kinase); PLK (polo kinase); RAN (Ran GTPase kinase); SCY1 (related to *S. cerevisiae* Scy1 kinase); ULK (Unc-51-like kinase); VPS (vacuolar protein sorting); WEE (small); IKS (Ira1 kinase suppressor); PDHK (pyruvate dehydrogenase kinase); PIKK (phosphatidyl inositol kinase-related kinase); RIO (right open reading frame); TTK (Tau Tubulin Kinase). **Other abbreviations**: Ank = contains ankyrin repeat domains; HU = HU sensitive; Camp = Camptothecin sensitive; DEO = DEO sensitive; Ben = benomyl sensitive; NaCl^s^ = growth inhibition in the presence of NaCl; Suc^s^ = growth inhibition in the presence of 1.5 M sucrose; NaCl^Rem^ = defects remediated on NaCl; Suc^Rem^ = defects remediated on sucrose; Colony color = Abnormal colony color; Brown = brown pigment produced by colonies/microcolonies; Yellow = yellow pigment production; Cell cycle = cell cycle defect; ^k^ = kinase domain deletion; V = variability in phenotype; Het = also recovered heterokaryons; UN = unnamed; ND = not determined; BH = best hit by BlastP. * = named in this study.

Notably, the above domain analysis identified 11 kinases with catalytic domains displaying only low similarity to the complete set of *S. cerevisiae*, *Caenorhabditis elegans*, *Drosophila melanogaster* and human kinases within the kinome database. We have called these novel kinases FfkA-K (filamentous fungal kinase) as they appear to be specific to filamentous fungi. Importantly recently available *A. nidulans* RNA-Seq data generated by the Caddick laboratory (http://www.aspgd.org/) indicates that apart from FfkH, which may represent a pseudogene, all Ffks are transcribed providing evidence that they are bona fide genes. Phylogenetic comparison of all *A. nidulans* kinase domains indicated that the FfkB (AN10819), FfkC (AN2373), FfkD (AN1789), FfkE (AN10869), FfkI (AN6768), FfkJ (AN6758) and FfkK (AN9302) are more closely related to each other than to other kinases, suggesting that they have evolved from a common ancestor (Figure 2, Figure S1 in File S1) and might represent a new fungal specific family of kinases. Of these kinases, FfkD and FfkE display 49.8% overall identity including a region of 28 amino acids in the predicted non-catalytic domain with 78.6% identity, suggesting that FfkD and FfkE represent a paralogous pair of kinases (Figure 2).

Phylogenetic comparison with other filamentous fungi indicated that, with the likely exception of *Laccaria bicolor*, Ffks are absent from the Basidiomycetes and that the presence of Ffk orthologues varies even within the Aspergilli (Figure S1 in File S1). For example, putative FfkF orthologues were identified in *A. terreus*, *N. fischeri*, *N. crassa* and *F. graminearum,* but were not identified in *A. fumigatus, A. oryzae, A. niger, A. flavus* or *A. clavatus* (Figure S1 in File S1). This comparison also revealed that kinases present in other filamentous fungi encoding catalytic domains that are related to FfkA (AN7986) also contain ankyrin repeat domains (Figure S1 in File S1). Similarly, FfkB, FfkC and kinases with related catalytic domains in other filamentous fungi also all contain ankyrin repeat domains (Figure S1 in File S1). The fact that these kinases share both similar regulatory sequences and catalytic domains suggests that they represent related kinases that likely have similar functions. Interestingly, *A. nidulans* lacks kinases belonging to the recently identified filamentous fungal specific FunK1 kinase family [9]–[11], [39]. Comparative genomic studies indicate that Funk1 kinases have been greatly expanded in a number of fungi including *Coprinopsis cinerea*, the Dermatophytes and the Paracoccidioides [9]–[11]. Although the function of Funk1 kinases has yet to be established, it has been suggested they might play roles in the developmental programs of more complex fungi [9]–[11], [39].

Comparison of the kinases encoded within the *A. nidulans*, *S. cerevisiae*, *S. pombe* and *N. crassa* genomes revealed additional notable differences. The most dramatic deviation from the normal repertoire of kinases was in the PIKK family of atypical protein kinases which have critical conserved functions in diverse cellular processes. For example, the Aspergilli and *N. crassa* each only encode a single Tor PIKK kinase whereas two Tor kinases are present in *S. pombe*, *S. cerevisiae* and humans (Figure S2 in File S1). Even more surprisingly, *A. nidulans* does not encode an orthologue of the Pik1 kinase which is essential in both *S. pombe* and *S. cerevisiae* where it has been implicated in the regulation of protein secretion, endocytosis, vacuolar dynamics and cytokinesis [40]. Other Aspergilli also lack a Pik1 orthologue although a Pik1 kinase (NCU10397) is encoded within the *N. crassa* genome (Figure S2 in File S1). One possibility is that the essential function of Pik1 in other organisms is carried out by the related Stt4 PIKK kinase in the Aspergilli.

As previously reported *A. nidulans* and *N. crassa* encode an expansion of histidine kinases (Figure S3 in File S1) [23], [26], [41]. An expansion of SRPK (serine-arginine protein kinases) kinases within the GMCG kinase group was also present in both *A. nidulans* and *N. crassa*. A similar expansion of SRPK kinases has recently been reported in Dermatophytes [9]. Members of this kinase family are known to phosphorylate serine within RS (arginine/serine rich) domains that are generally found in splicing factors and other proteins involved in mRNA maturation [42]. Whereas *S. pombe* and *S. cerevisiae* each encode a single SRPK family member, Dsk1 and Sky1 respectively, *A. nidulans* encodes 7 and *N. crassa* encodes 5 predicted SRPK kinases (Figure S4 in File S1). We named the *A. nidulans* Dsk1/Sky1 orthologue SrpkA^Dsk1^ and the other SRPK kinases SrpkB-G. Importantly, analysis of RNA-Seq data (http://www.aspgd.org/) indicates that all 7 *A. nidulans* SRPKs and 15 histidine kinases are expressed.

### Generation of *A. nidulans* Kinase Deletion Null Mutants

**Figure 3:**
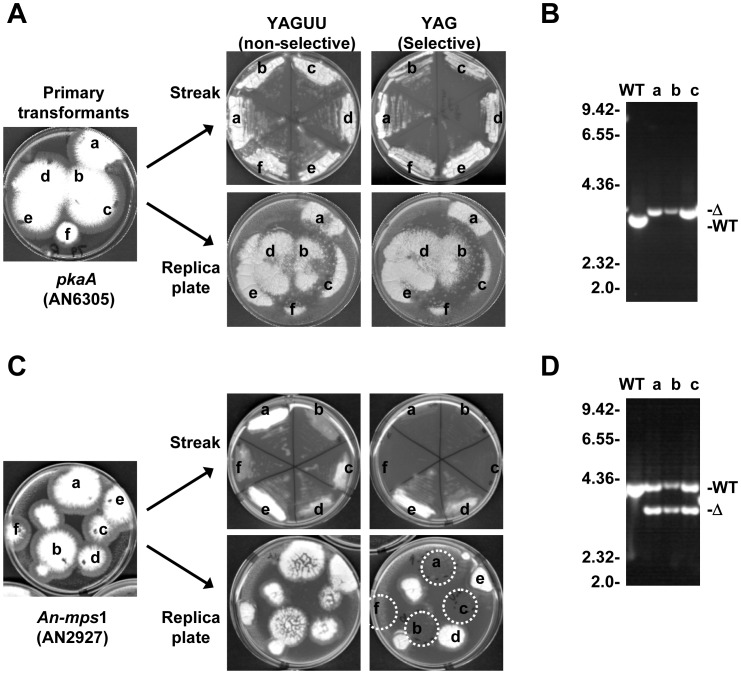
Identification of essential and non-essential kinases by heterokaryon rescue . (A) A primary transformation plate for the *pkaA* kinase deletion construct (left). Conidia from the indicated colonies were tested for their ability to form colonies on selective (YAG) or non-selective (YAGUU) media by either replica streaking or replica plating using a velvet disk. Growth of all 6 tested transformants on selective media suggests that *pkaA* is non-essential. (B) Diagnostic PCR indicates that 3 tested transformants are haploid nulls confirming that *pkaA* is non-essential. (C and D) As for (A and B) but showing analysis of an essential gene. Following transformation of the *An-mps1* deletion construct, conidia from 4 of 6 tested transformants were unable to form colonies on selective media, strongly suggesting that *An-mps1* is essential. Diagnostic PCR indicates that 3 of these heterokaryotic transformants contain both the wild type and deleted allele, confirming that *An-mps1* is essential.

To illustrate the utility of the gene deletion constructs and provide tools useful to the research community, we systematically generated gene knock out strains of each *A. nidulans* kinase. Kinase deletion constructs were transformed into a *pyrG*89, Δ*kuA^ku70^* recipient strain selecting for growth on media lacking uridine and uracil. The Δ*kuA^ku70^* genetic background of this strain facilitated a high yield of construct targeting via homologous recombination [43]. For each gene deletion multiple primary transformants were tested to determine if the respective kinase was essential or non-essential using the heterokaryon rescue technique [35]. This technique is based on the ability of *A. nidulans* to form heterokaryons containing two genetically distinct nuclei within a common cytoplasm. Following deletion of an essential kinase, primary transformant colonies can maintain a heterokaryotic state with cells containing transformed (kinase -) nuclei providing PyrG function, and untransformed (*pyrG-*) nuclei providing the essential kinase function. However, this heterokaryotic state is not maintained during asexual conidiospore (conidia) production as these conidia are uninucleate. Such heterokaryotic colonies therefore generate 2 types of uninucleate conidia from the heterokaryons. These conidia can be distinguished on selective media on which *pyrG-* cells do not form germ tubes, contrasting the *pyrG+* cells which can form germ tubes but subsequently arrest with the kinase deleted phenotype. An important part of this analysis is that conidia streaked on non-selective media should display the kinase deleted phenotype at the same frequency as on selective media. Contrasting this, following deletion of a non-essential kinase, conidia from primary transformants should grow similarly on selective or non-selective media (Figure 3A). We found that replica plating of primary transformants using a velvet disk greatly expedited this analysis and allowed both essential and non-essential kinases to be readily identified by heterokaryon rescue (Figure 3A and C). Generated heterokaryons or haploid nulls were genotypically confirmed by diagnostic PCR using primers situated external to the targeting sequence of each kinase deletion construct to determine the presence of the wild type and/or deleted kinase allele [38] (Figure 3B and D). Using this analysis, 25 kinases (19.5%) were defined as essential because haploid nulls could not be propagated on standard YAG selective media but could be maintained as heterokaryons.

This deletion protocol worked with high efficiency although obtaining verified deletion strains for large kinase genes required screening a greater number of transformants and for the 7703 bp *phkA* (AN3102) and 6183 bp *An-stt4* (AN4278) kinases, we did not originally obtain verified kinase deletion strains. We reasoned that the size difference between the 1732 bp *pyrG* ^Af^ marker cassette and such large genes decreased the efficiency of gene replacement by homologous recombination. Thus we eliminated the kinase activity of An-Stt4 and PhkA by deleting a smaller region of the gene including their entire kinase domains.

The FfkJ (AN6758), FfkK (AN9302) and PkpB (AN9461) kinases were identified after the inception of this study and deletion strains for these kinases have not been generated here. Kinase deletion strains for the other 128 kinases have been deposited at the FGSC (http://www.fgsc.net/Aspergillus/KO_Cassettes.htm) and are characterized below.

### Phenotypic Analysis of Non-essential Kinase Mutants

#### Non-essential kinase mutants displaying general growth defects

**Figure 4:**
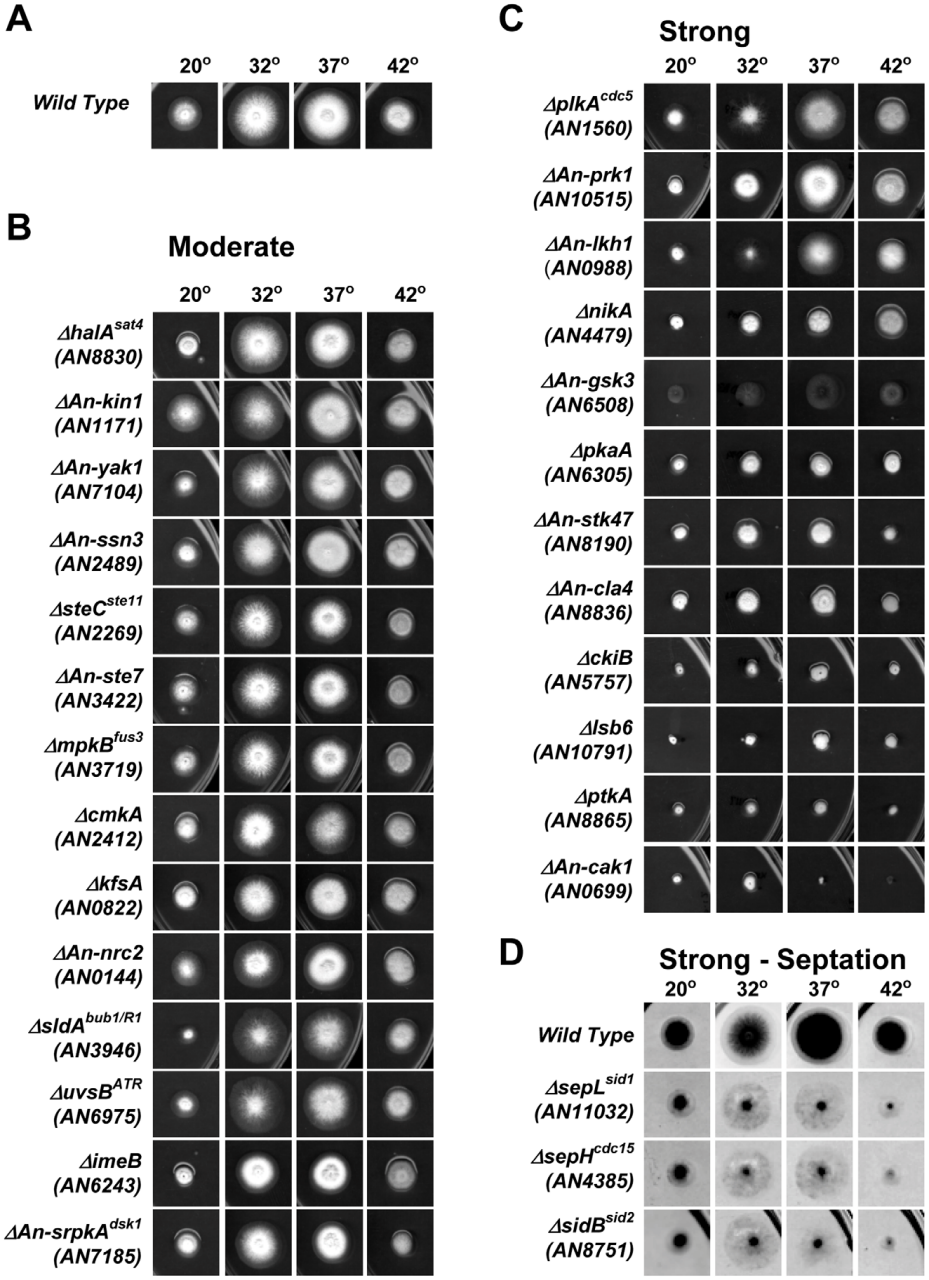
Kinase deletion mutants with vegetative growth defects. Shown is colony formation of the indicated strains after 2 days at 32°, 37° or 42°, or 5 days at 20°. (A) An isogenic wild type control. (B) Kinase deletion mutants with a moderate but reproducible colony growth defect. (C) Kinase deletion mutants with a strong colony growth defect. (D) Septation deficient kinase deletion mutants display a strong growth phenotype characterized by extremely poor conidiation. Colonies are shown as negatives to more clearly visualize the hyphae.

We analyzed colony formation of non-essential kinase mutants at 20°, 32°, 37° and 42° to determine if these kinases had roles in vegetative growth and development. This identified 29 mutants, including 14 previously uncharacterized kinases, with growth defects and/or poor conidiation (Figure 4). Of these kinase mutants, 14 displayed moderate but reproducible growth defects while 15 displayed strong growth defects. In some cases the growth defect was so severe that we also initially obtained heterokaryons following transformation (Table 1). However, as haploid nulls for these kinases could be propagated as genetically stable haploids on selective media they were classified as non-essential.

#### *A. nidulans* encodes 3 septation initiation network kinases

Deletion of either the *sepH^cdc15^* (AN4385) or *sidB^sid2^* (AN8751) kinases, which are required for septation, resulted in the expected phenotype of extremely poor conidiation with little effect on radial growth as indicated by colony diameter [44]–[46]. Unlike *S. cerevisiae*, *S. pombe* encodes a third kinase called Sid1 which is required for septation as part of the septation initiation network (SIN). AN11032 (previously called AN8033 in version 2 of the *A. nidulans* genome) encodes a predicted Sid1 orthologue which, although not experimentally studied, has been termed sepL [47] (Figure S5 in File S1). The colonies of *sepL* ^sid1^ mutants were phenotypically identical to the *sepH^cdc15^* and *sidB^sid2^* mutants, strongly suggesting that SepL^Sid1^ is required for septation and conidiation (Figure 4D). To confirm this, we stained the cell walls of *sepL* ^sid1^ mutant germlings with calcofluor which revealed an almost complete absence of septa even in cells with over 32 nuclei (Figure S5B and C in File S1). Thus, SepL^sid1^ is a third kinase required for septation in *A. nidulans*.

#### Kinase mutants with defects in development and secondary metabolism

**Figure 5:**
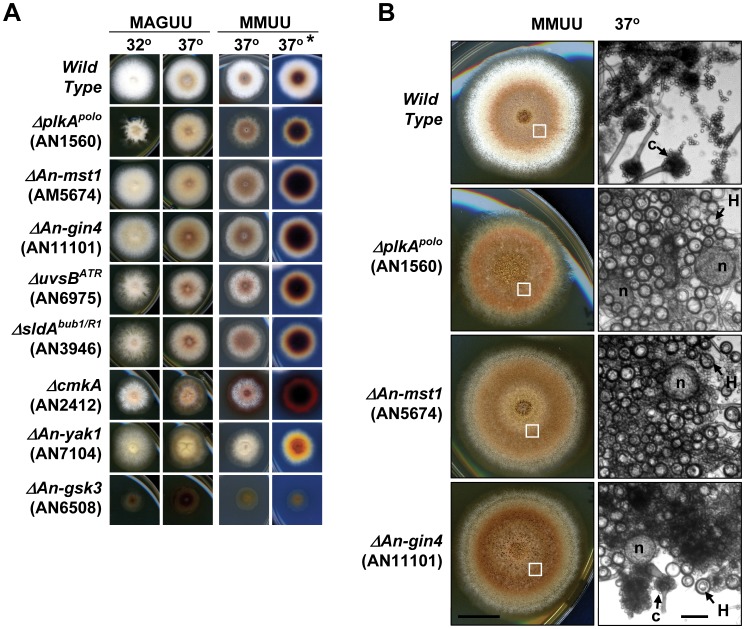
Kinase deletion mutants with developmental defects. (A) Colony color after 3 days growth of the indicated strains and conditions. * Images for the MMUU series at 37° are also shown from the underside of the colony to more clearly show pigments produced by the colonies. (B) The left column shows colonies point inoculated with 10^4^ spores after 4 days growth at 37°. The *ΔplkA^polo^*, *ΔAn-mst1* and *ΔAn-gin4* strains produce few asexual conidia but show advanced sexual development. The right column shows cells collected from the indicated region of each colony at higher magnification. In contrast to the asexual conidiophores (c) and enormous numbers of asexual conidia of the wild type colony, the *ΔplkA^polo^* and *ΔAn-mst1* mutant colonies contain numerous Hulle cells (H) surrounding apparent nascent cleistothecia (n). The *ΔAn-gin4* mutant also contained Hulle cells and apparent nascent cleistothecia, although more conidiophores were apparent relative to the other mutants. Bar ∼ 100 µm.

**Figure 6:**
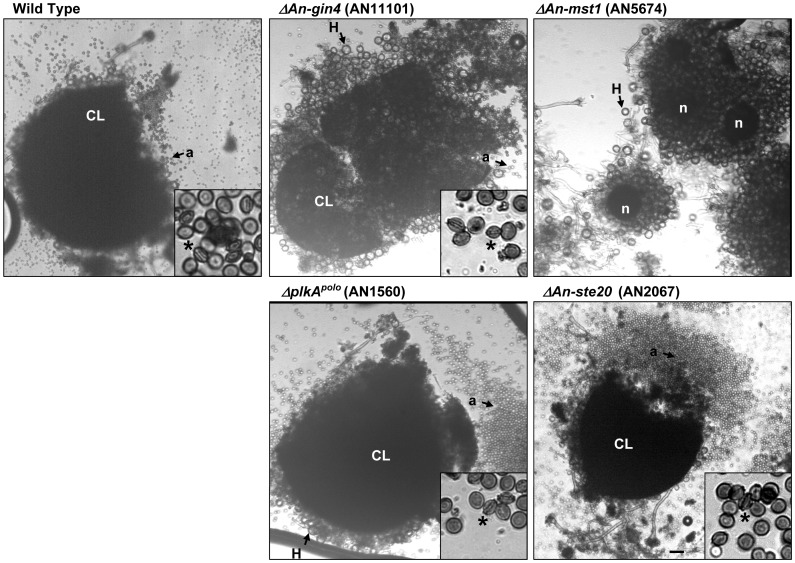
Cleistothecia and ascospore formation in the indicated kinase mutants. Shown are micrographs taken of cells collected from point inoculated colonies after 13 days growth at 37° on minimal media. By this time wild type colonies and the Δ*An-gin4*, Δ*plkA* and Δ*An-Ste20* mutant colonies had formed cleistothecia (CL) containing red pigmented ascospores (a). Contrasting this, Δ*An-mst1* mutant colonies had formed only immature nascent cleistothecia (n) which did not contain ascospores. The insets show the ascospores at higher magnification with the * indicating example ascospores orientated such that their bivalve morphology is apparent. Bar ∼ 100 µm.

As *A. nidulans* is homothallic (self-fertile) sexual development can occur in the absence of a mating partner [4]. The onset of sexual development is often indicated by changes in colony color due to the formation of yellow Hulle cells surrounding red pigmented fruiting bodies called cleistothecia and the production of secondary metabolites secreted into the medium [4], [48]. After 3 days growth 8 non-essential kinase mutants formed colonies displaying a color distinctly different from the white parent strain (Figure 5A). These color differences correlated with a dark appearance of the underside of colonies grown on minimal media at 37° (Figure 5A), conditions which promote sexual development. Further examination indicated that the aberrant colony color of the *plkA* (AN1560), *An-gin4* (AN11101) and *An-mst1* (AN5674) mutants was associated with visible morphological hallmarks of sexual development, as previously reported for *plkA* mutants [49]. By 4 days, wild type colonies contained large numbers of asexual conidiophores with little or no evidence of sexual development. Contrasting this, *plkA*, *An-gin4* and *An-mst1* mutant colonies displayed reduced asexual development but increased sexual development as indicated by vast numbers of Hulle cells which often surrounded pigmented nascent cleistothecia (Figure 5B). However, whereas *plkA* and *An-gin4* mutants continued sexual development and formed mature cleistothecia containing ascospores, *An-mst1* mutants arrested in the early stages of cleistothecia formation and only produced immature nascent cleistothecia which did not contain ascospores (Figure 6).

For other kinase mutants the aberrant colony color was not associated with visible morphological signs of sexual development. *An-yak1* (AN7104) mutant colonies were unique in that they produced a yellow pigment whereas the other mutants produced a brown pigment which was most obvious for the slow growing Δ*An-gsk3 (*AN6508) colonies grown on rich media (Figure 5A). These Δ*An-gsk3* colonies produced very few asexual spores as has recently be shown for *F. graminearum gsk3* mutants [14]. The secreted brown pigment of the *cmkA* (AN2412) mutant was most dramatic and spread forming a halo around the colony edge (Figure 5A). A similar pigment was produced by the *uvsB^ATR^* (AN6975) and *sldA^bub1/R1^* (AN3946) mutants but apparently in lower quantities.

The so called MAPK (mitogen activated protein kinase) module consists of the SteC^Ste11^ (AN2269), Ste7 (AN3422) and MpkB^fus3^ (AN3719) kinases and functions in a phosphorylation cascade required for sexual development [50], [51]. Consistent with this, the 3 MAPK module mutants displayed an identical moderate growth defect (Figure 4B, Figure S6 in File S1) and, as has previously been reported, did not form cleistothecia [4], [51]–[53]. However, although the MAPK module is predicted to be activated by the An-Ste20 (AN2067) kinase [50], *An-ste20* mutants displayed a distinct phenotype from the MAPK module mutants. Most notably, unlike the MAPK module mutants, *An-ste20* mutants were able to undergo sexual development resulting in the formation of mature cleistothecia containing ascospores (Figure 6). MAPK module mutants and *An-ste20* mutants also displayed significant differences in the apparent production of secondary metabolites. For example, when colonies were grown on minimal media, *An-ste20* mutants secreted a brown pigment on their surface which was not produced by the 3 MAPK module mutants (Figure S6B in File S1). Interestingly, production of this surface brown pigment by *An-ste20* mutants was apparent on minimal media but not rich media (Figure S6A–B in File S1), suggesting a role for An-Ste20 in nutrient sensing. Contrasting this, when the MAPK module mutants where grown on rich media in top agar cultures they secreted a dark pigment into the medium which was not apparent in *ΔAn-ste20* or wild type cultures inoculated at the same density (Figure S6C in File S1). The production of this pigment by the MAPK module mutants is likely a result of their arrest at an early stage of sexual development [4], [51]–[53]. These data are consistent with the regulation of sexual development and secondary metabolite production by the MAPK module [4], [51]. However, while the 3 MAPK module kinases are required for sexual development, the predicted upstream An-Ste20 kinase is not always required indicating that the regulation of this pathway is more complicated than predicted.

### Drug Sensitivities of Kinase Deletion Mutants

#### Kinases required for the cellular response to genotoxic stress

**Figure 7:**
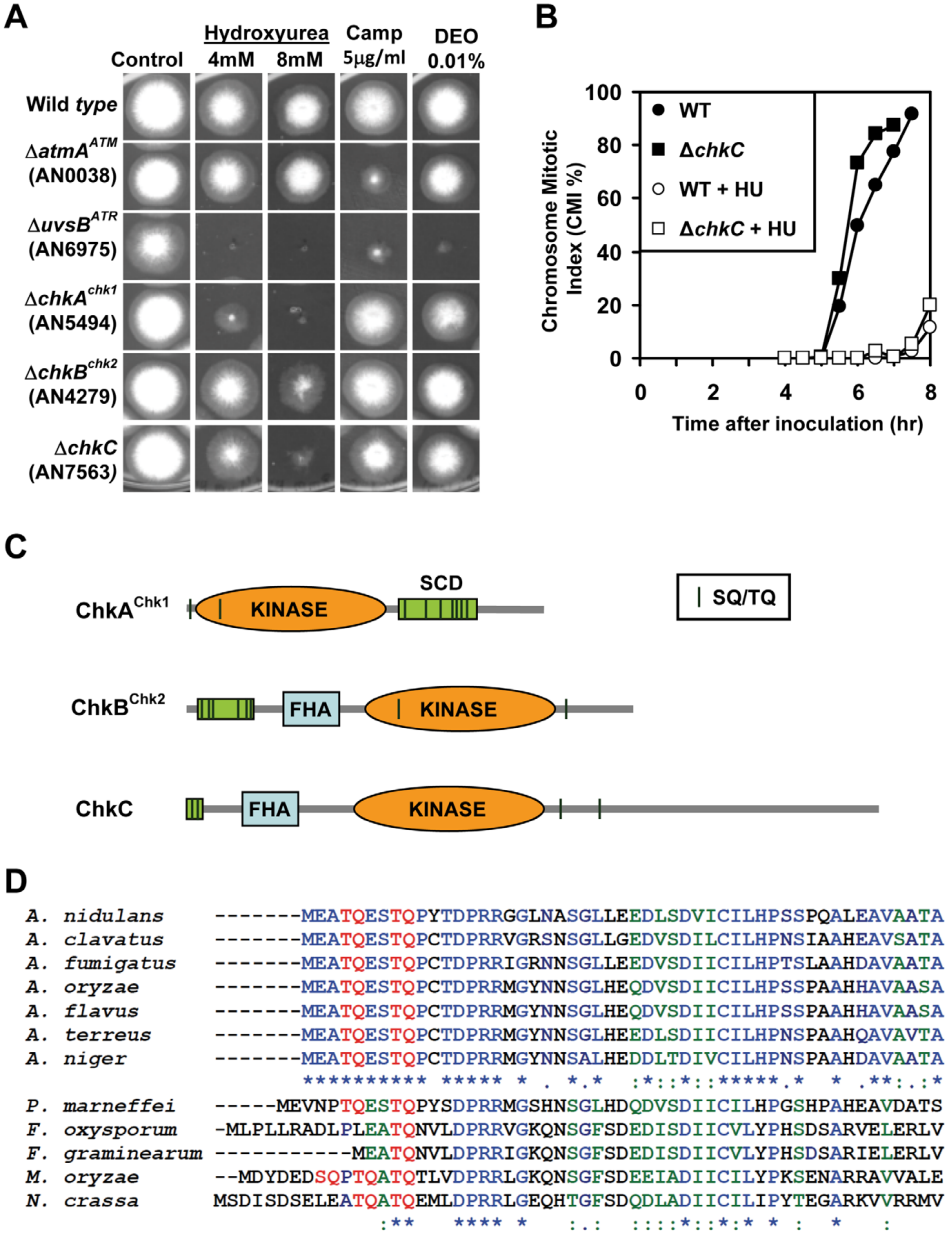
*A. nidulans* kinase deletion mutants with sensitivities to genotoxic agents. (A) Colony formation of the indicated strains with or without the indicated genotoxic agents. Images were taken after 2 days at 32° except for the 8 mM HU series which is after 3 days. (B) *chkC* mutants do not enter mitosis prematurely in the presence of HU. Wild type and *ΔchkC* conidia were inoculated in the presence or absence of 10 mM HU and the chromosome mitotic index (CMI) of DAPI stained cells determined at each time point. Benomyl (2.4 µg/ml) was included in the media to help maintain a mitotic arrest once cells entered mitosis [118]. (C) Schematic diagrams showing the domain structure of the indicated kinases. (D) ClustalW alignment (http://workbench.sdsc.edu/) of the N-terminals of ChkC kinases. Identical (*), conserved strong groups (:), and conserved weak groups (.) are indicated. Di-peptide SQ and TQ motifs are indicated in red. Accession numbers for the sequences are; *A. nidulans* (AN7563; EAA62143), *A. clavatus* (ACLA_072560; EAW14223), *A. fumigatus* (Afu2g14920; XP_755825), *A. oryzae* (AO090012000405; BAE60587), *A. flavus* (EED54418), *A. terreus* (ATEG_07832; EAU32094), *A. niger* (An15g03280; CAK42403), *P. marneffei* (EEA23567.1), *F. oxysporum* (FOXB_01143, EGU88344), *F. graminearum* (Fg00433), *M. oryzae* (EHA53924), *N. crassa* (NCU02751; Mus-59).

In response to genotoxic stress the conserved PIKK kinases ATR and ATM phosphorylate serine or threonine within the SQ/TQ cluster domains (SCDs) of the downstream effector kinases Chk1 and Chk2 [31]. In *A. nidulans* these 4 kinases are encoded by *uvsB^ATR^* (AN6975), *atmA^A^™* (AN0038), *chkA^chk1^* (AN5494) and *chkB^chk2^* (AN4279) [54], [55]. As previously reported, *uvsB* ^ATR^ mutants were sensitive to a wide variety of genotoxic agents including the DNA alkylating agent DEO, the DNA double strand break inducing agent camptothecin and the DNA replication inhibitor HU [56], [57]. Contrasting this, the *atmA* ^A^™ mutant was only strongly sensitive to camptothecin, consistent with ATM kinase functions being more specific for double strand DNA breaks [31], [58], [59] (Figure 7). Both the *chkA^chk1^* and *chkB^chk2^* effector kinase mutants were sensitive to HU but not the DNA damaging agents tested (Figure 7). As HU sensitivity was previously reported for *chkA^chk1^* but not *chkB^chk2^* mutants [59], we tested the viability of these mutants at different concentrations of HU (Figure S7 in File S1), confirming their HU sensitivity.

Notably, in addition to the *uvsB^ATR^*, *chkA^chk1^* and *chkB^chk2^* mutants, deletion of the previously uncharacterized ChkC (AN7563) kinase also resulted in HU sensitivity (Figure 7A). Importantly the HU sensitivity of *chkC* mutants was independent of the presence of the Δ*kuA^ku70^* mutation (Figure S8 in File S1). To determine if Δ*chkC* HU sensitivity was due to loss of cell cycle checkpoint regulation, we germinated cells in the presence or absence of 10 mM HU and followed their entry into mitosis. The presence of HU delayed mitotic entry of *chkC* mutants in a manner similar to wild type cells (Figure 7B). This indicates that the checkpoint which delays mitotic entry in the presence of unreplicated DNA is intact and that the HU sensitivity of *chkC* mutants is due to a defect in another aspect of the DNA damage response. Although ChkA^Chk1^, ChkB^Chk2^ and ChkC are each CAMK kinases, the kinase domain of ChkC is more closely related to ChkB^Chk2^ (35.7%) than to ChkA^Chk1^ (28.6% identity) (Figure S9 in File S1). Moreover, ChkC displays structural similarity to Chk2 kinases and is an orthologue of mus-59 which encodes a second Chk2 related kinase in *N. crassa* [60]. Similar to ChkB^Chk2^ and other Chk2 kinases, ChkC contains a forkhead associated domain (FHA) which potentially mediates phosphorylation dependent protein-protein interactions (Figure 7C, Figure S9 in File S1). ChkC kinases are present in other filamentous ascomycetes but are absent from basidiomycetes including *U. maydis* which remarkably does not encode any Chk2 related kinases [61]. Notably, the Aspergillus ChkC kinases each contain three conserved SQ/TQ sites, two of which are clustered together in the N-terminus including at least one that is conserved in the more distantly related filamentous ascomycetes *N. crassa, Penicillium marneffei*, *Fusarium oxysporum*, *F. graminearum* and *Magnaporthae oryzae* (Figure 7D). Thus ChkC kinases encode an N-terminal SCD domain containing potential sites of phosphorylation by the ATM and/or ATR kinases and in *A. nidulans* ChkC is involved in the cellular response to replicative stress.

#### Kinases responsive to osmotic stress

**Figure 8:**
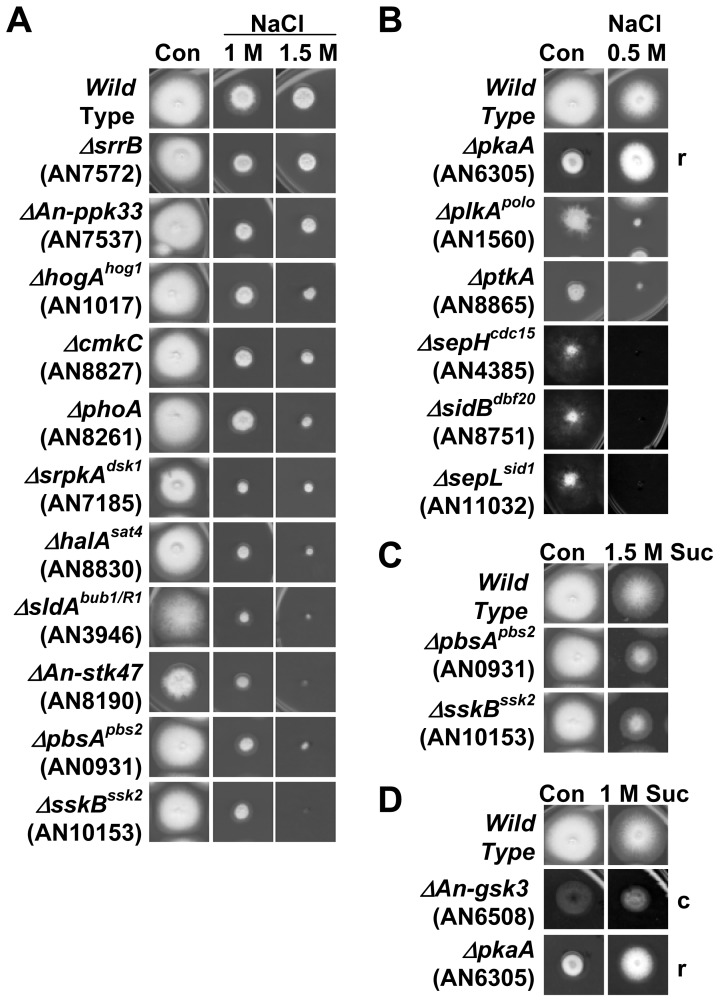
Kinase deletion mutants whose growth is reduced or enhanced by osmotic agents. Shown are the indicated deletion strains grown with or without the indicated concentrations of NaCl or sucrose. Growth is after 3 days at 32° except for the 1.5 M NaCl series which is after 4 days. Mutants were classified as having (A) moderate sensitivity to osmotic stress or (B) strong sensitivity to osmotic stress. (C) Kinase mutants whose colony formation was inhibited by 1.5 M sucrose. (D) Kinase mutants whose defects were partially remediated by sucrose. r = remediation of growth, c = remediation of conidiation.

The growth of 16 kinase mutants was reproducibly inhibited in the presence of 1 or 1.5 M NaCl (Figure 8). This included null alleles of the previously characterized osmotic stress response kinases *hogA^hog1^* (AN1017), *pbsA^pbs2^* (AN0931) and *sskB^ssk2^* (AN10153), which encode orthologues of the budding yeast HOG-MAPK (high-osmolarity glycerol response mitogen activated protein kinase) pathway [30]. In addition to reduced growth in the presence of NaCl, *pbsA^pbs2^* and *sskB^ssk2^* mutants also displayed reduced growth in the presence of 1.5 M Sucrose (Figure 8C). However, increased osmolarity did not inhibit growth of *tcsB* ^sln1^ histidine kinase (AN1800) or *steC* ^ste11^ (AN2269) kinase mutants, even though these kinases encode HOG-MAPK pathway orthologues, confirming previous studies in *A. nidulans* [30]. Also confirming a previous report, growth of *halA* kinase (AN8830) deleted strains was inhibited in the presence of NaCl [62].

Our analysis also identified mutants whose growth was reproducibly inhibited to a similar or greater level as the above bona fide osmotic stress response kinase mutants. These included deletion mutants of the *cmkC* (AN8827), *phoA* (AN8261), *sldA^bub1/R1^* (AN3946), *plkA^polo^* (AN1560), *ptkA* (AN8860), *sepH^cdc15^* (AN4385), *sidB^sid2^* (AN8751) and *srrB^rim15^* (AN7572) kinases for which sensitivity to osmotic stress has not been previously reported (Figure 8). Although the *plkA^polo^* and *ptkA* mutants displayed strong growth defects in the absence of osmotic stress, other kinase mutants with similar strong growth defects were not similarly inhibited (Figure 8B). We also identified 4 previously uncharacterized kinases, SrpkA^Dsk1^ (AN7185), An-Stk47 (AN8190), An-Ppk33 (AN7537), and SepL^Sid1^ (AN11032), whose deletion resulted in growth inhibition in the presence of NaCl (Figure 8). Notably, the 3 SIN kinase mutants each displayed marked sensitivity to osmotic stress (Figure 8B).

Interestingly, the strong growth defect of the *pkaA* (AN6305) mutant was significantly remediated by increased osmolarity (Figure 8B and D). Along with PkaB (AN4717), PkaA is one of two cAMP-dependent protein kinase catalytic subunits in *A. nidulans*. As *pkaA* is partially redundant with *pkaB* [63], one possibility is that under conditions of osmotic stress *pkaB* is up-regulated thereby suppressing the lack of *pkaA*. Sucrose also partially remediated the poor conidiation of the *An-gsk3* (AN6508) mutant even though it did not improve the poor radial growth of this mutant (Figure 8D, Figure S10 in File S1).

#### *A. nidulans* encodes a single Bub1/BubR1 kinase required for the spindle assembly checkpoint

The majority of organisms encode two proteins related to the Bub1 spindle assembly checkpoint kinase. Humans encode the Bub1 and BubR1 kinases, while budding and fission yeast encode a Bub1 kinase and the related Mad3 protein which lacks a kinase domain [64]. A recent study has found that this complex organization of paralogues is the result of 9 distinct gene duplications combined with the subfunctionalization of the duplicated genes [65]. As part of this remarkable example of parallel evolution, the kinase function of one paralogue is almost always lost. This can occur by either kinase domain deletion as for the Mad3 proteins, or by mutation of the kinase domain resulting in a pseudokinase as has been argued for human BubR1 [65]. In *A. nidulans* SldA^Bub1/R1^ (AN3946) is the only member of the Bub1/BubR1/Mad3 family and similarly only a single orthologue is present in other Aspergilli and *N. crassa* (Figure S11D in File S1). This indicates that *bub1* gene duplication has interestingly not occurred in these filamentous ascomycetes and suggests that SldA^Bub1/R1^ must perform all Bub1/BubR1/Mad3 functions. Consistent with this SldA^Bub1/R1^ contains all the functional domains of the Bub1/BubR1/Mad3 family including a kinase domain which is more similar to the Bub1 kinase than the BubR1 pseudokinase (Figure S11B and C in File S1).

As expected given its function in the spindle assembly checkpoint, deletion of *sldA^Bub1/R1^* resulted in marked sensitivity to the microtubule poison benomyl as previously shown (Figure S11A in File S1) [66], [67]. Interestingly however, *sldA^bub1/R1^* mutants also displayed moderate growth defects and osmotic stress sensitivity which was not displayed by Δ*mad1* spindle assembly checkpoint mutants (Figure S11A in File S1). This suggests that SldA^Bub1/R1^ has functions in addition to its role in the spindle assembly checkpoint.

### Functional Analysis of Essential Kinases by Heterokaryon Rescue

*A. nidulans* offers the advantage that essential gene phenotypes can be readily studied using heterokaryon rescue [35]. Using this technique we determined the phenotype of cells lacking the function of 23 of the 25 essential kinases. We were unable to determine the phenotype of cells lacking An-Aurora (AN5815) or An-Mps1 (AN2927) function as the respective heterokaryons did not apparently generate conidia containing the deleted kinase allele. As both An-Aurora and An-Mps1 encode predicted cell cycle regulators, it is possible that severe cell cycle defects in conidiophores are preventing the generation of the respective kinase deleted conidia from the heterokaryons. For the other essential kinases, kinase deleted cells were identified by their ability to undergo limited growth on selective media, contrasting the kinase wild type conidia from the same heterokaryon which did not form germ tubes due to lack of *pyrG* function. The phenotypes of cells lacking essential kinases were further classified based on their nuclear and cellular morphology as well as the extent to which they were able to grow.

#### Kinase mutants which terminally arrest as very short germlings

**Figure 9:**
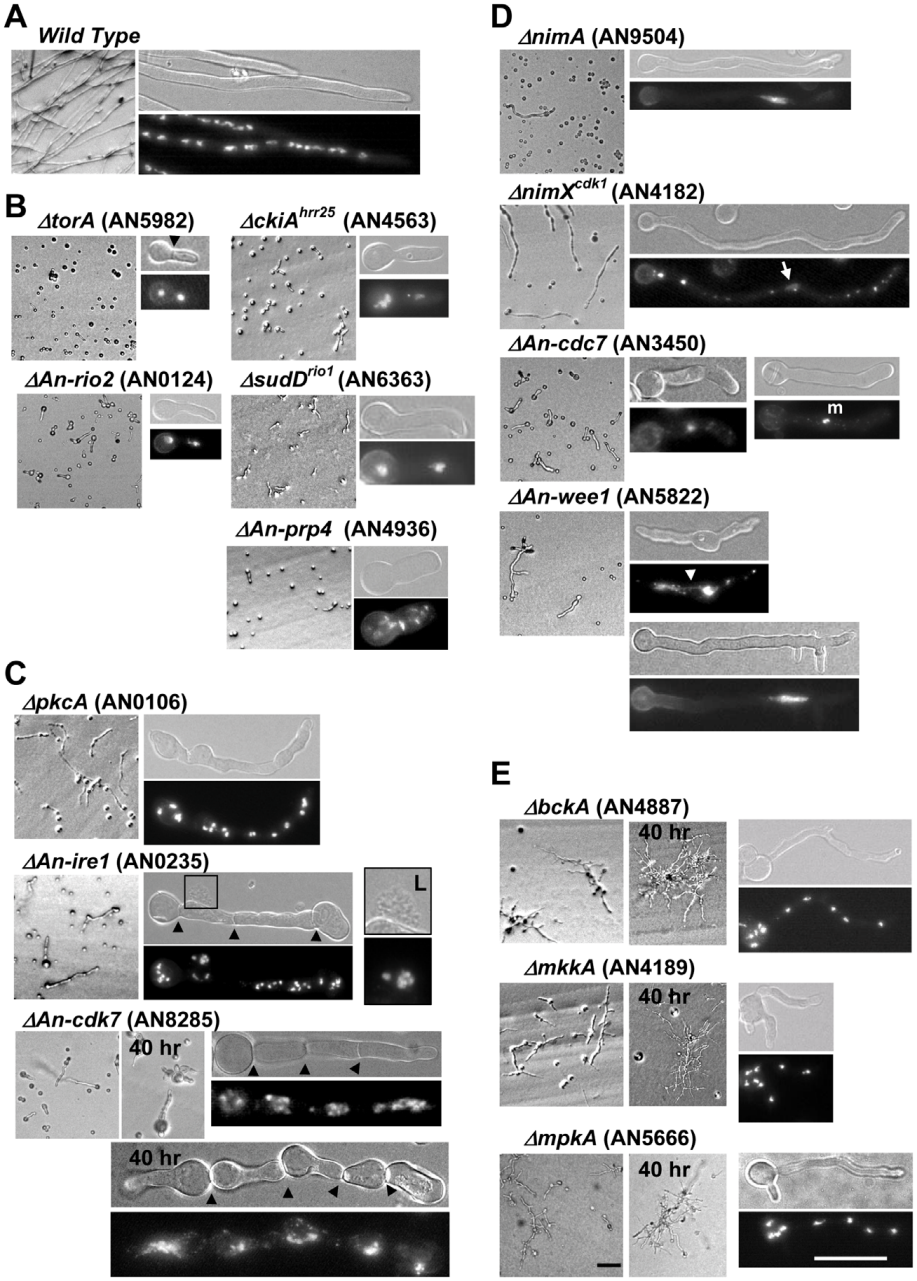
Null phenotypes of essential kinase mutants. Uninucleate conidia from heterokaryons grown in media selective for growth of only the kinase deleted cells. Shown are representative cells grown on plates or on coverslips for DAPI staining. Images are micrographs after ∼18 hr growth at 32° unless indicated. (A) An isogenic strain wild type for all kinases. Kinase nulls where classified based on the predominant phenotype as (B) arresting growth as short germlings, (C) arresting growth as swollen cells, (D) undergoing limited polarized growth in the absence of nuclear division to give a cell cycle arrest phenotype, or (E) arresting growth as thin branched germlings displaying an abnormal morphology. Black arrowheads indicate septa. L = lysed cells. Note that *An-cdc7* mutants displayed only a single nucleus which in ∼24% of cells appeared mitotic (m). The white arrow distinguishes the single interphase nucleus in the *nimX^cdk1^* mutant from the DAPI staining mitochondria. The white arrowhead indicates DNA stretched between 2 masses of DNA in cells lacking the An-Wee1 kinase. Bars ∼ 50 µm.

Kinase deletions with the most severe phenotypes formed only short germlings after two days growth indicating that they play vital roles in essential cellular processes (Figure 9B). Among these was the *An-prp4* (AN4936) mutant which encodes an orthologue of the *S. pombe* Prp4 kinase required for mRNA splicing [68]. The *torA* (AN5982), *ckiA^hrr25^* (AN4563), *sudD^rio1^* (AN6363) and *An-rio2* (AN0124) mutants also arrested growth as short germlings (Figure 9B). Interestingly, based on the functions of their budding yeast orthologues, each of these 4 kinases is predicted to play a key role in ribosomal biosynthesis [69]–[71]. While this suggests that the terminal phenotype of each of these mutants is due to a defect in ribosomal biosynthesis, these kinases also have other predicted cellular functions [72]–[74]. Interestingly, cells lacking TorA kinase function displayed a second phenotype in which short germlings with 2 or fewer nuclei frequently displayed septa, a phenotype never seen in wild type cells (Figure 9B). This phenotype is seemingly unrelated to known functions of yeast Tor kinases as regulators of protein synthesis, ribosome biogenesis, autophagy and the transcriptional response to cellular stresses or nutrient availability [70], although chemical inhibition of Tor kinase in *Podospora anserina* interestingly also leads to increased septation [75].

#### Kinases with cell cycle functions

In *A. nidulans* preventing cell cycle progression blocks nuclear division but does not prevent polarized growth, causing cells to arrest as elongated germlings containing single nuclei [76]. Depending on the cell cycle stage in which the gene has an essential function, mutants arrest with either a never in mitosis (NIM) phenotype or a blocked in mitosis (BIM) phenotype. As expected, we observed a NIM phenotype following deletion of the essential NIMA (AN9504) kinase [28], [77], [78] (Figure 9D). A single interphase nucleus was also apparent in *nimX^cdk1^* (AN4182) deleted cells but this nucleus was often obscured by brightly staining mitochondrial DNA (Figure 9D) [77]. To more clearly examine these nuclei, we deleted *nimX^cdk1^* in a Histone H1-mCherry strain. This confirmed that *nimX^cdk1^* nulls arrest with a NIM phenotype and indicated that during a prolonged interphase arrest nuclei continued to enlarge resulting in a diffuse nuclear chromatin signal (Figure S12A in File S1).

Cells lacking An-Cdc7 (AN3450) kinase function contained a single nucleus which in ∼24% of cells was mitotically condensed (Figure 9D). This is significantly higher than the mitotic index of wild type cells which is typically ∼5%. We confirmed that such Δ*An-cdc7* cells were mitotic by deleting *An-cdc7* in a GFP-Tubulin and Histone H1-mCherry strain which revealed that cells often displayed monopolar spindles (Figure S12B in File S1). The predicted An-Cdc7 binding partner NimO^Dbf4^ is required to initiate DNA replication and NimO^Dbf4^ mutants are also defective in the activation of the checkpoint which prevents mitotic entry before completion of DNA replication [79]. Examination of *An-cdc7* mutants indicated that they displayed an identical phenotype to *nimO^dbf4^* mutants resulting in cells entering mitosis with unreplicated DNA (Figure S12C in File S1), as occurs for Cdc7 mutants in other systems [80]. Thus although An-Cdc7 is essential for DNA replication, cells display a mixed NIM and BIM phenotype because cells which fail to initiate DNA replication are also defective in checkpoint regulation over mitotic entry.

The primary function of Wee1 kinases to carry out an inhibitory phosphorylation of the Cdk1 kinase is not essential in *A. nidulans* as non-Wee1-phosphorylatable *nimX^cdk1^* mutants are viable [81]. However, we surprisingly found that An-Wee1 (AN5822) has an essential function which is thus likely distinct from its role in phosphorylating NimX^cdk1^ (Figure 9D). Cells lacking An-Wee1 predominantly arrested with branched germ tubes and displayed a high frequency of nuclei with an irregular size, shape and distribution along germ tubes (Figure 9D; Figure S12D in File S1). Nuclei were also often connected by strings of stretched DNA caused by a failure in mitotic DNA segregation. Enlarged nuclei of cells lacking An-Wee1 also often displayed multiple spindles consistent with these nuclei being polyploid and containing multiple spindle pole bodies (Figure S12D in File S1). These phenotypes are consistent with lethal defects in cell cycle regulation. However why Wee1 is essential in *A. nidulans* and *U. maydis* [82] but is not essential in *N. crassa* [16] is unclear.

#### Kinase mutations which effect cellular integrity

**Figure 10:**
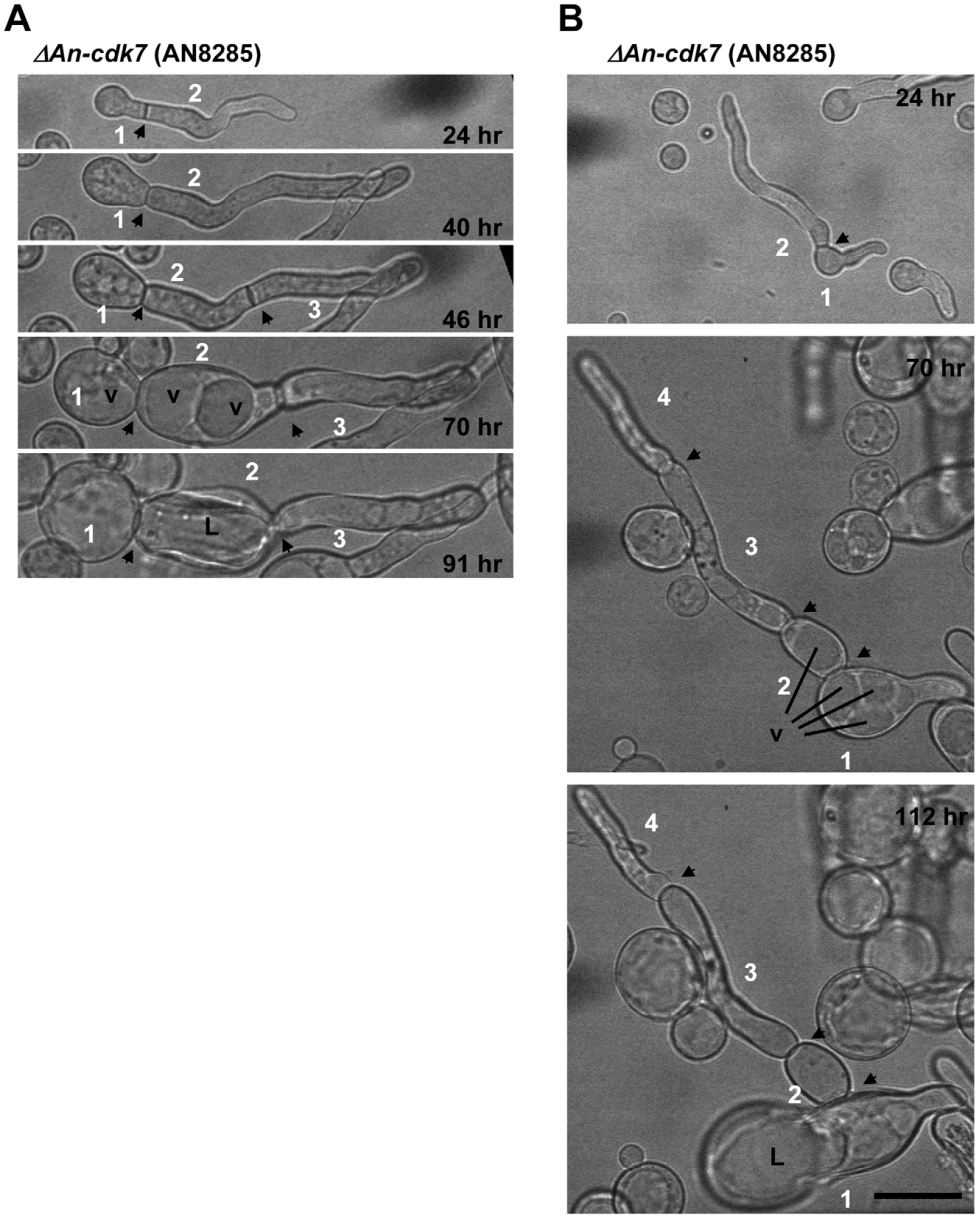
The extreme swelling of *An-cdk7* mutants correlates with a massive enlargement of vacuoles. (A and B) Time lapse images of conidia inoculated from the heterokaryon in media selective for growth of only the *An-cdk7* deleted cells. Cells separated by septa (arrowheads) are numbered sequentially as they form. Cells initially appear normal but undergo extreme swelling which appears to be potentially mediated by an enlargement of vacuoles (v). Note that septa restrict swelling and following lysis (L) appear to seal the junction between adjacent cells. Bar ∼ 50 µm.

Deletion of the PkcA (AN0106), IreA (AN0235) or An-Cdk7 (AN8285) kinases resulted in differing degrees of cellular swelling (Figure 9C) which was not suppressed by increased osmolarity (data not shown). The most striking swollen phenotype was for *ΔAn-cdk7* mutants which were so swollen by 40 hr that it was difficult to determine if cells had originated from an individual spore as they often appeared like individual cells lined up in a row (Figure 9C). Time lapse brightfield microscopy revealed that *ΔAn-cdk7* germlings initially appeared normal but, after septation, underwent drastic swelling during further growth (Figure 10). Interestingly cells separated by septa underwent swelling to different extents with the most pronounced swelling generally occurring in sub-apical cells. In these cells the extent of cellular swelling corresponded with the size of the vacuoles which were often massively enlarged (Figure 10). Another hallmark of this phenotype was that the septal cell wall did not appear to expand in the same manner as the rest of the cell wall leading to the cell restrictions observed between the swollen cells.

As previously reported, cells lacking PkcA kinase function underwent swelling and occasionally lyzed (Figure 9C) [83], [84]. PkcA is also predicted to regulate a basic three kinase module consisting of BckA (AN4887), MkkA (AN4189) and MpkB (AN5666) which is analogous to the *S. cerevisiae* cell wall integrity pathway [50]. Cells deleted for the BckA, MkkA or MpkB kinases each displayed an identical phenotype in which mutants formed microscopic branched colonies with an irregular morphology after 40 hr (Figure 9E). As the lethal phenotype of the equivalent budding yeast mutants is remediated by high osmolarity [85], we determined if this was also the case for the *A. nidulans* mutants. The presence of either 1 M sucrose or 1 M NaCl had a dramatic effect whereby *bckA*, *mkkA* and *mpkB* mutants now formed conidiating colonies after two days, although *pkcA* mutants still failed to form colonies under these conditions (Figure S13 in File S1). We conclude that BckA, MkkA and MpkB function in a pathway analogous to the *S.cerevisiae* cell wall integrity pathway and the PkcA likely has other functions in addition to regulating this pathway.

The swelling displayed by cells lacking IreA kinase function was most pronounced at the cell tips (Figure 9C). This swelling is presumably related to the well established function of this family of kinases as mediators of the unfolded protein response [86]. In addition to *pkcA*, *ireA* and *An-cdk7*, the *An-prp4* and *An-cdc7* kinase mutants also displayed moderate cellular swelling although this was not the primary defect of these mutants (Figure 9).

#### Kinase mutants defective in polarized growth or vesicular trafficking can form microcolonies

**Figure 11:**
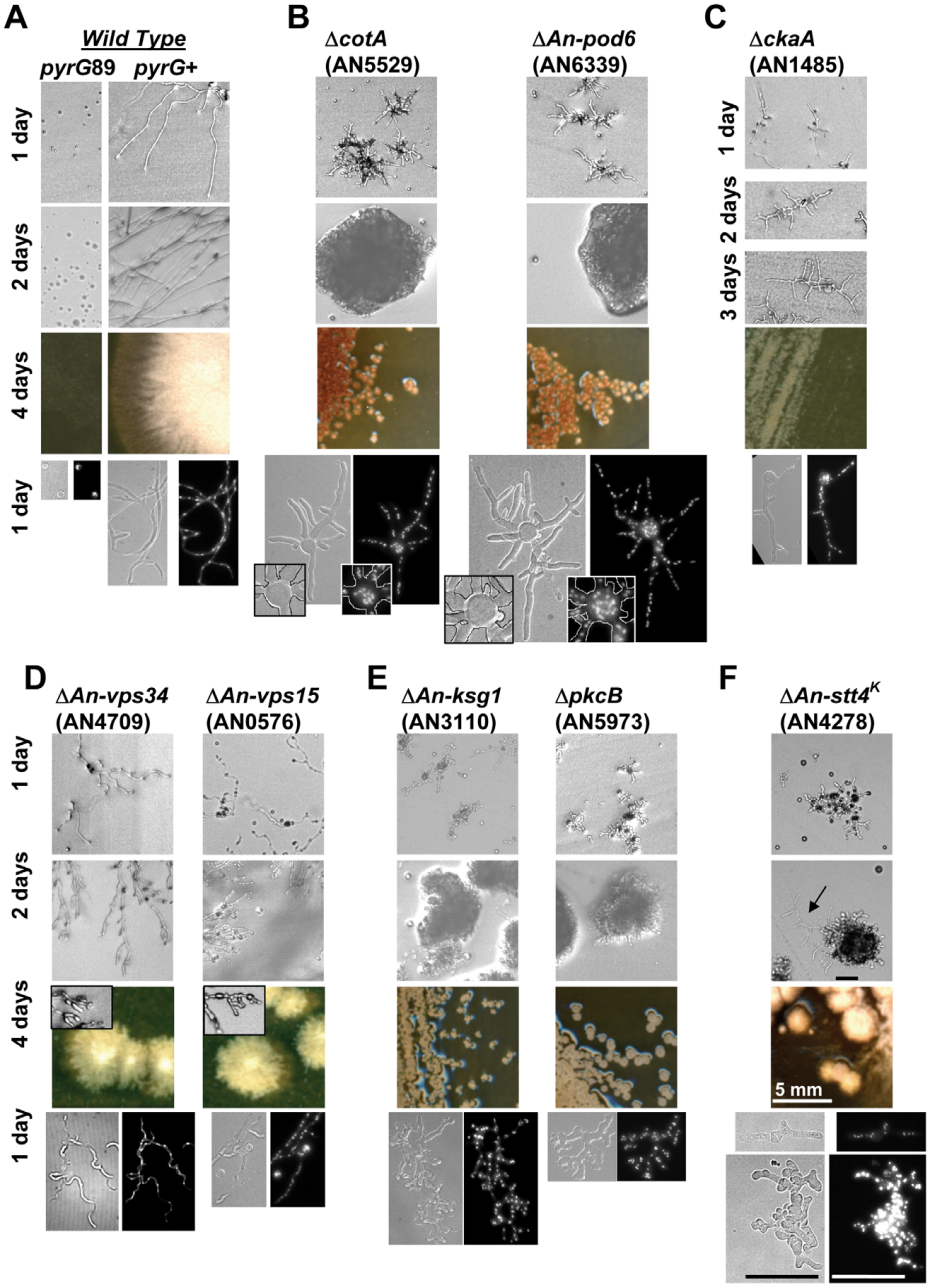
Essential kinase deletion mutants which form microcolonies. Uninucleate conidia from heterokaryons were streaked on selective media (top 3 rows) or inoculated and fixed for DAPI staining (bottom row). After 4 days growth at 32^o^ these kinase mutants formed microcolonies which could not be propagated as genetically stable haploids by streaking. (A) Wild type *pyrG*+ and *pyrG89* strains. (B) *An-pod6* and *cotA* nulls display an identical brown microcolony phenotype. Insets show over 5 germ tubes emerging from an enlarged spore body indicative of a defect in the establishment of polarized growth. (C) *An-cka1* nulls initiate polarized growth normally but arrest as small microcolonies. (D) The *An-vps15* and *An-vps34* nulls display an identical phenotype. Insets show highly segmented cells at colony edges. (E) The *An-ksg1* and *pkcB* nulls initiate a nearly identical irregular pattern of growth and branching before arresting as microcolonies. (F) The *An-stt4* kinase domain deletion mutant displays a range of phenotypes. The arrow indicates a section of a microcolony which appears to have resumed normal growth. The bottom row shows 2 cells from the same field which have grown to different extents and display distinctly different DNA content. Bar ∼ 50 µm.

A less severe phenotype which permitted the formation of microcolonies was displayed by 8 different kinase deletion mutants (Figure 11). We classified these kinases as essential as mutants only formed microcolonies that did not produce viable spores. Most strikingly, cells lacking either CotA (AN5529) or An-Pod6 (AN6339) kinase function formed morphologically identical microcolonies which secreted a brown pigment (Figure 11B, Figure S14C in File S1). Both *An-pod6* and *cotA* nulls displayed up to 8 germ tubes emanating from a swollen spore (Figure 11B), consistent with the known function of CotA in maintaining cellular polarity [87]. *N. crassa* Cot-1 is orthologous to CotA and the phenotype of *cot-1* mutants can be remediated under conditions of high osmolarity [88]. However 1 M sucrose or 1 M NaCl did not remediate the microcolony phenotype of either *cotA* or *An-pod6* deleted cells, although it interestingly decreased pigment production (Figure S14A and B in File S1). The identical polarity defect of *cotA* and *An-pod6* mutants is consistent with the functions of the orthologous kinases in *N. crassa* and *S. pombe* to regulate polarized growth [89]–[91]. This has been best studied in *S. pombe* in which Nak1, the likely orthologue of An-Pod6, functions upstream of Orb6, orthologous to CotA, as part of the MOR (morphogenesis Orb6) network which regulates actin location to promote polarized growth [92]. Following the MOR regulated location of actin to sites of polarized growth, the *S. pombe* Cka1 casein kinase II regulates a subsequent step in polarized growth [93]. The role for casein kinase II in regulating polarized growth is conserved in *S. cerevisiae* [94] and also likely *A. nidulans* as cells lacking An-Cka1 (AN1485) arrested with a microcolony phenotype (Figure 11C). Based on the *S. pombe* data and the phenotypes of the *A. nidulans* mutants, we predict that An-Pod6 and CotA function in a network analogous to the MOR and that An-Cka1 performs a yet to be defined role in polarized growth.

Cells lacking the *An-vps15* (AN4709) or *An-vps34* (AN0576) kinases formed morphologically identical microcolonies consistent with their common function in endosomal trafficking (Figure 11D) [95]. Although these microcolonies displayed highly segmented and branched tip cells, the initial growth of these *vps* kinase mutants was relatively normal. Contrasting this, both *An-ksg1* (AN3110) and *pkcB* (AN5973) kinase deleted cells initiated an irregular pattern of growth and branching before arresting as microcolonies (Figure 11E). This phenotypic similarity is consistent with budding yeast Sc-Phk2 (orthologous to An-Ksg1), regulating Sc-Ypk1 (orthologous to PkcB) in a sphingolipid-mediated signaling pathway involved in endocytosis [96]. It was also noticeable that the early growth morphology of *An-stt4* mutants was similar to that of *An-ksg1* and *pkcB* mutants, suggesting that the predicted An-Stt4 function in sphingolipid biosynthesis contributes to this phenotype (Figure 11F). However, budding yeast Stt4 has many functions [97] and *An-stt4* mutants displayed phenotypes in addition to their growth defect. We consistently observed that as Δ*An-stt4* cells grew individual nuclei stained more brightly for DNA, suggesting a cell cycle defect potentially related to the G2/M arrest phenotype of budding yeast *stt4* mutants (Figure 11F; the DAPI stained cells are from the same field) [98]. However, while many *An-stt4* mutant cells arrested growth as short branched cells with an apparently abnormal DNA content, others formed aconidiate microcolonies (Figure 11F). Microscopic examination of the *An-stt4* mutants indicated that colony sectors with defective polarized growth occasionally resumed somewhat normal growth (Figure 11F, arrow). This suggests that mutations which suppress the *An-stt4* mutant phenotype might be arising spontaneously and permitting further growth. Similarly, the microcolonies of the *vps* kinase mutants also occasionally formed sectors with improved growth suggesting the appearance of suppressor mutations in these cells (Figure S15 in File S1). Similar spontaneous suppressors have recently been isolated from several other *vps* mutants [95], suggesting that such suppression is a relatively frequent event in this class of mutants. This highlights the importance of maintaining essential gene deletions with a relatively weak arrest phenotype as heterokaryons in which there will be no selection for such suppressor mutations.

## Discussion

Utilizing gene deletion constructs generated here for the majority of *A. nidulans* genes, we have generated kinase deletion strains for 128 kinases and identified phenotypes for 68 of these mutants. One advantage of large scale genetic analysis is that mutants generated in the same genetic background have the potential to be phenotypically grouped as likely regulators of the same process when similar phenotypes are uncovered. Given that kinases often function together in signaling pathways, this phenotypic grouping provided experimental evidence that previously uncharacterized kinases function in the SIN, cell wall integrity and MOR kinase signaling pathways, as well as in pathways regulating vesicular trafficking and the cellular response to replicative stress. This analysis also confirmed previously known kinase functions in the MAPK module and the HOG-MAPK pathway. Importantly however the phenotypes of some kinase mutants differed from those defined for orthologues in other systems. This is not unexpected as, for example, it is known that kinases which are essential in one organism can be non-essential in others. This holds true also within the filamentous fungi as orthologues of the essential *A. nidulans* kinases, An-Prp4, Wee1, NIMA, An-Vps15 and An-Cka1, are non-essential in *N. crassa* [16].

### Kinases Specific to Filamentous Fungi

Of considerable interest *A. nidulans* encodes 11 Ffks (filamentous fungal kinases) unrelated to the recently identified Funk1 fungal specific kinase family. Seven of these Ffks, including the likely paralogues FfkD and FfkE, appear to have originated from a common ancestor. Potential functional redundancy among related Ffks might in part explain why we did not identify phenotypes for these mutants. However, as Ffks are present in some Aspergilli but not others, it is likely that these kinases have very specialized functions which might be difficult to define. It will be important to determine if Ffks are active kinases or if they potentially encode pseudokinases which may compete with other kinases for substrates. Interestingly, among the sequenced Aspergilli, *A. terreus* is unique in that it encodes a member of the Funk1 kinase family (ATEG_09985). In striking contrast to its absence from other Aspergilli however, kinases highly related to ATEG_09985 are present in many other fungi with the most similar (74% query coverage at an E-value of 0.00) occurring in the entomopathogenic fungus *Metarhizium anisopliae*. This interesting phylogenetic distribution suggests that the presence of the ATEG_09985 Funk1 kinase in *A. terreus* might be the result of horizontal gene transfer. A common theme among filamentous fungal specific kinases such as the Ffks and Funk1 kinases is that they have often been expanded or lost during the evolution of individual filamentous fungal species. This suggests that these kinases are potentially involved in regulating the diverse array of developmental programs which occur in these organisms [39]. In the long term, defining the function of filamentous fungal specific kinases is important as orthologues present in pathogenic fungi are potential candidates for the development of specific antifungal agents.

Also noteworthy are the significant expansions of histidine kinases and SRPK kinases in *A. nidulans* relative to the yeasts *S. cerevisiae* and *S. pombe*. The expansion of histidine kinases in filamentous fungi has been suggested to help these organisms respond to a broad range of environmental stimuli [23], [26], [41] although these signaling pathways are presently poorly understood. The expansion of SRPK kinases and potential SRPK pseudokinases in filamentous fungi is not understood [9]. However, as SRPK kinases are regulators of mRNA processing, one possibility is that the SRPK expansion reflects an increased importance of splicing in filamentous fungi relative to *S. cerevisiae* whose genes contain few introns. Consistent with this, *S. cerevisiae* does not encode a Prp4 mRNA splicing kinase which is essential in *A. nidulans* and in *S. pombe* [68]. As was the situation for the Ffks, many *A. nidulans* histidine kinases and SRPKs are closely related and potentially function in a redundant manner.

Intriguingly, we have identified ChkC as a second Chk2 like kinase present in the filamentous ascomycetes. Although ChkC is orthologous to *N. crassa* Mus-59, their mutations cause sensitivity to different types of genotoxic stress, ChkC to replicative stress, and Mus-59 to DNA double strand breaks [60]. Thus, as for the Chk1 and Chk2 effector kinases [31], [59], [60], the requirement for ChkC orthologues in response to genotoxic stress varies in different organisms. In addition, it is known that functional redundancy exists between effector kinases and it will be important to determine if ChkC has overlapping functions with ChkA^Chk1^ and/or ChkB^Chk2^.

### Kinases Involved in Maintaining Cellular Integrity

Our analysis indicates that the BckA, MkkA and MpkA kinases function in a pathway analogous to the cell wall integrity pathway of *S. cerevisiae*, as the lethality of the respective mutants was suppressed by high osmolarity. Although the PkcA kinase is predicted to function upstream of the cell wall integrity pathway, cells lacking *pkcA* displayed a more severe phenotype which was not suppressed by high osmolarity. This suggests that PkcA has regulatory targets in addition to the cell wall integrity pathway, possibly at the growing cell tip or at septa where PkcA localizes [83], [84].

After an initial period of normal polarized growth, cells lacking An-Cdk7 underwent dramatic swelling which correlated with a massive enlargement of vacuoles. An-Cdk7 is part of a kinase family including budding yeast Kin28 and fission yeast Mcs6 which regulate RNA pol II transcription and in many organisms have a second function as a CDK activating kinase (CAK) [99]. In *S. pombe mcs6* mutants fail to separate following septation, most likely as a consequence of changes in their transcriptional profile [100]. It is thus interesting that in *An-cdk7* mutants the most severe swelling occurred in sub-apical cells after septum formation. Thus one possibility is that An-Cdk7 regulates transcriptional changes in sub-apical cells following septation. Potentially sub-apical cells lacking An-Cdk7 may express an inappropriate profile of genes leading the pronounced vacuolar and cellular swelling in these cells.

Our results regarding orthologues of the budding yeast HOG-MAPK pathway which responds to osmotic stress are consistent with previous studies [30]. Importantly however, our findings also suggest unknown functions for the CmkC, PhoA, SldA^Bub1/R1^, PlkA^Polo^, PtkA, SrpkA^Dsk1^, An-Stk47, An-Ppk33 and SrrB^Rim15^ kinases in the cellular response to osmotic stress.

The pronounced osmotic stress sensitivity of the *sepL* ^sid1^, *sepH^cdc15^* and *sidB^sid2^* septation deficient SIN kinase mutants supports the concept that septa help maintain cellular integrity by compartmentalizing cells as they grow and explore the environment. Contrasting the lack of septation in the SIN mutants, *torA* kinase mutants underwent precocious septation. This suggests that in addition to their well established essential functions, Tor kinases play a yet to be defined role as negative regulators of septation in filamentous fungi [75].

### Kinases Regulating Sexual Development and Secondary Metabolite Production

Relative to an isogenic wild type control strain, 14 kinase mutants displayed altered secondary metabolite production and/or sexual development. However, it is important to note that all strains in this study, and most strains historically used for experimental study of *A. nidulans*, contain a mutation in the VeA protein (*veA1*), a key coordinator of secondary metabolite production and sexual development [4], [101]. Thus in future studies it will be important to determine if kinase mutant phenotypes are altered in a *ve*A+ background. For example, it is known that *imeB* (AN6243) kinase mutants display increased sexual development in a *veA*+ but not a *veA*1 background [102]. Consistent with this the Δ*imeB* mutant in this study did not display advanced sexual development.

As expected the *steC^ste11^*, *ste7* and *mpkB^fus3^* MAPK module mutants displayed few signs of sexual development and did not form cleistothecia [4], [51]–[53]. Most surprisingly however, cells lacking the predicted upstream An-Ste20 PAK (p21 activated kinase) kinase formed mature cleistothecia containing ascospores. Phenotypic differences between *F. graminearum* FgMAPK module mutants and the *FgSte20* mutant have also been recently reported [14]. Thus it will be interesting to determine if in these and other filamentous fungi Ste20 does indeed activate the MAPK module and/or if it has additional regulatory targets involved in developmental regulation. Notably however, deletion of An-Mst1, a GCK (germinal center kinase) STE20 related kinase, resulted in incomplete sexual development. Further, An-Mst1 orthologues in *N. crassa* and *F. graminearum* also display developmental defects [14], [103], [104]. Thus one interesting possibility is that An-Mst1, whose kinase domain is similar to An-Ste20 (2e−54), contributes to the regulation of the MAPK module during sexual development in filamentous fungi.

Similar to *An-mst1*, the *An-gin4* and *An-plkA* kinase mutants also displayed a decrease in asexual spore production and the early onset of sexual reproduction. This confirms a recent finding for PlkA [49] and suggests that these kinases negatively regulate the developmental switch from asexual to sexual reproduction. PlkA is a polo like kinase and members of this family have developmental functions in mammals, flies and worms [105]–[108]. Therefore, understanding how PlkA negatively regulates sexual development remains an important area for future study. One possibility based on the known cell cycle related functions for PlkA and Gin4 kinase family members [109], [110], is that the developmental defects of *An-gin4* and *plkA* mutants reveal an important link between cell cycle control and development. The recent finding that *F. graminearum gin4* mutants also display developmental defects provides further evidence that Gin4 is a regulator of sexual development in filamentous fungi [14]. Interestingly however, whereas *An-gin4* mutants undergo early sexual development and form ascospores, *F. graminearum gin4* mutants undergo incomplete sexual development [14].

Varying degrees of pigment production without obvious signs of sexual development was observed following deletion of the An-Pod6, CotA, UvsB^ATR^, SldA^Bub1/R1^, CmkA, An-Yak1 or An-Gsk3 kinases. Interestingly one of the functions of the budding yeast An-Gsk3 orthologue, Rim11, is to induce the expression of meiosis specific genes [111]. Thus one possibility for the increased pigment production in *An-gsk3* mutants is that altered meiotic gene expression has uncoupled secondary metabolite production from sexual development. An alternative explanation for increased pigment production is that the normal response to environmental stimuli is defective in certain kinase mutants. It is thus informative that brown pigment production by *An-pod6* and *cotA* mutants was suppressed by increased osmolarity. Thus pigment production of these mutants may be related to the proposed function for *N. crassa cot-1* in an environmental stress response [112]. It will also be interesting to determine if the yellow pigment produced by *An-yak1* mutant colonies is related to the glucose sensing function observed for the *S. cerevisiae* orthologue Yak1 [113].

### Kinases Essential for Growth

We identified 25 kinases which had an essential function preventing the null allele being propagated as a genetically stable haploid. Importantly, we were able to further classify these kinases based on the terminal phenotype of mutants determined by heterokaryon rescue. Kinase mutants with a predicted essential function in vital processes such as ribosome biogenesis displayed the strongest growth arrest while kinase mutants with predicted functions in maintaining polarized growth or vesicular trafficking were able to form very slow growing microcolonies. For the latter, maintaining the deleted allele in heterokaryons removed selection for faster growing cells and prevented the selection of mutations which suppressed the microcolony phenotype. At least two features of these microcolonies likely contribute to the relatively frequent appearance of these suppressor mutations. Firstly the increased number of nuclei in these cells increases the probability of these mutations arising and, secondly, the microcolony arrest phenotype can relatively easily be compensated for by mutations in other genes as demonstrated for *vps20, vps23, vps27* and *vps36* mutants [95].

Our finding that the IreA kinase is essential strongly suggests that the unfolded protein response is an essential process in *A. nidulans*. *S. cerevisiae* Ire1 locates to the ER and responds to the accumulation of unfolded proteins by regulating the transcriptional activation of genes encoding proteins involved in protein folding [114]. In filamentous fungi, it has been suggested that protein secretion during polarized growth increases the demand for protein folding thereby triggering the unfolded protein response [115]. Thus the accumulation of unfolded proteins which would normally be secreted might explain the swollen phenotype of *ireA* mutants. Interestingly this apparent requirement for the unfolded protein response, or how it is regulated, varies in different fungi as Ire1 is essential in *A. nidulans*, *A. niger* and *F. graminearum* but non-essential in *A. fumigatus, S. pombe* and *S. cerevisiae* [14], [115]–[117]. An alternative explanation is that Ire1 has an unknown essential function/s in some but not all fungi. Similar arguments can be made for other kinases which are essential in some, but not all filamentous fungi.

### Kinases Essential for the Cell Cycle

At least four *A. nidulans* kinases have essential cell cycle functions, the previously uncharacterized An-Cdc7 kinase and the NIMA, NimX^Cdk1^ and Wee1 kinases. It is also likely that the An-Aurora and An-Mps1 kinases have essential cell cycle functions although we were unable to define the phenotype of cells lacking these kinases. Our finding that An-Wee1 has an essential function that is likely independent of NimX^Cdk1^ regulation was most surprising. However, although *nimX^cdk1^* mutants which cannot be phosphorylated by An-Wee1 are viable, they lack functional interphase cell cycle checkpoints and are highly sensitive to DNA damage or perturbed DNA replication [81], [118]. When DNA replication is inhibited in these *nimX^cdk1^* mutants they undergo aberrant mitosis resulting in irregular nuclei often connected by strands of DNA [56]. Interestingly, Δ*An-wee1* cells frequently displayed a highly similar phenotype during otherwise unperturbed cell cycles. In this regard it is interesting that recent unbiased screens have revealed that human Wee1 has an additional function during DNA replication [119], [120]. If this were also the situation in *A. nidulans*, cells lacking An-Wee1 might take longer to complete replication and additionally fail to activate the checkpoint preventing mitotic entry in response to the incompletely replicated DNA. Thus, independent defects in DNA replication and checkpoint regulation might explain why cells lacking An-Wee1 undergo aberrant mitosis.

### Perspectives

Consistent with the vast array of cellular functions carried out by kinases, *A. nidulans* kinase mutants display a wide range of phenotypes from almost no growth to relatively subtle or no effects on vegetative growth. It is likely that further characterization of this kinase deletion set will reveal additional new phenotypes. Utilization of a similar deletion set of *A. nidulans* protein phosphatases [19], together with the kinase deletion mutants generated in this study, should advance the understanding of protein phosphorylation in filamentous fungi. Together with the expanding database of *A. nidulans* mutants (http://www.aspgd.org/), phenotypes defined here should aid in the classification of mutants of as yet uncharacterized genes generated using deletion constructs made available as part of this project. This expanding mutant library should help provide the basis for systems level biology in *A. nidulans*.

## Materials and Methods

### Deletion Construct Generation

Deletion construct primers were designed using software as described with modified criteria [34]. For most genes the deletion construct was designed such that *pyrG^Af^* would replace the target gene within 30 bp +/− of the start codon (89.3%) and stop codon (85.4%). When primers could not be designed in this region, the region before or after the start and stop codon was increased to 75 bp. Design of deletion construct primers was successful for 10,079 genes (95.4%) and primers for each deletion construct are listed in Table S1. The *pyrG* ^Af^ cassette was amplified from plasmid pCDS60 using primers CDS164 and CDS165. Synthesis of the 5′ and 3′ flanking region of each gene was carried out by a Biomek NX robot (Beckman) from *A. nidulans* genomic DNA using LA Taq (TaKaRa) as described [34]. To generate full length deletion constructs, yeast strain FY834 was transformed with the 3′ and 5′ flanking pieces, the *pyrG* ^Af^ cassette and plasmid pRS426 (digested with XbaI and EcoRI), and yeast DNA prepared as described [34]. Gene specific primers 5f and 3r were used to amplify the full length deletion constructs. For some kinases, deletion constructs were generated by fusion PCR [37], [38].

### Bioinformatic and Phylogenetic Analysis

Orthologues of *A. nidulans* kinases present in other Aspergilli were identified by BLAST search at the AspGD [122] (http://www.aspgd.org/). Kinase domains were identified by BLAST comparison with the Salk Institute’s kinome database (http://kinase.com/ [20]) or using a Batch CD search at the NCBI [121]. Phylogenetic analysis was carried out using ClustalW (http://workbench.sdsc.edu/or http://www.phylogeny.fr). Trees were visualized using MEGA version 5 [123] or Biology Workbench (http://workbench.sdsc.edu/).

### Generation of Kinase Deletion Strains

Deletion constructs were transformed into strain SO451 (*pyrG*89; *wA*3; *argB*2*; ΔnkuA^ku70^::argB pyroA*4; *sE*15 *nirA*14 *chaA*1 *fwA*1) which contains the *nkuA^ku70^* gene deletion to facilitate high frequency homologous recombination [43]. Transformation of 20 µl of SO451 protoplasts with 2 µl of the robotically generated deletion construct generated sufficient transformants for further analysis for most kinases. When necessary, the deletion construct DNA was concentrated or re-amplified prior to transformation in order to obtain sufficient number of transformants. Transformed protoplasts were plated onto YAG media containing 1 M sucrose, to maintain osmotic stability, and lacking uridine and uracil to allow selection for integration of the *pyrG* ^Af^ marker. Six independent transformants for each construct were initially tested by either “replica streaking” or replica plating onto YAG and YAGUU media using a velvet disk. Growth on YAGUU but not YAG suggested that deletion of the kinase was lethal and had resulted in the formation of heterokaryons. This was further assessed by microscopic examination of the selective YAG plates looking for the presence of both germinated (*pyrG*+; Δ*kinase*) and ungerminated (*pyrG*89; *kinase*+) cells which is characteristic of heterokaryons formed after deletion of an essential gene. A similar frequency of phenotypically similar *pyrG*+; Δ*kinase* germlings on the YAGUU plates, along with germlings displaying wild type growth (*pyrG*89; *kinase*+), provided additional evidence that an essential kinase had been deleted. For such putative heterokaryons, agar plugs were excised from the colony edge of the primary transformants and propagated for further analysis by transfer onto a YAG plate or stored at −80° [35]. For kinase deletion constructs which did not generate heterokaryons, three independent transformants were streaked to single colony prior to further analysis. After the final streak, conidia were inoculated in YG liquid media to generate mycelia which were lyophilized for DNA extraction as described [124]. For putative heterokaryons, mycelial excised from the growing colony edge was inoculated into YG media, grown and similarly processed.

Transformants were tested for site specific integration of the deletion construct by diagnostic PCR using primers (Table S1) situated external to the targeting sequence for each kinase deletion construct. In most cases the size difference between the wild type and null allele was sufficient to distinguish them on a gel. When necessary alleles were distinguished using restriction enzymes which cut within *pyrG^Af^* of the deleted allele but did not cut the wild type allele. The presence of both the wild type and null allele in transformants successfully streaked on selective media indicated that diploids had formed during the transformation and these were discarded. Confirmed non-essential kinase haploid deletion strains and heterokaryons of essential kinases, have been deposited at the FGSC (http://www.fgsc.net/Aspergillus/KO_Cassettes.htm) and are listed in Table S2.

Deletion of *An-cdc7*, *nimX^cdk1^* and *An*-*wee1* was also carried out by transformation of HA365 (*pyrG*89*; pyroA*4 *ΔnkuA::argB*; *argB/argB2*; *Histone H1-mCherry::pyroA^Af^*; *GFP-tubA*) selecting and verifying heterokaryons as above.

### Phenotypic Analysis of Kinase Deletion Strains

Two independent deletion strains for each non-essential kinase were phenotypically characterized in an initial test for colony growth at 20°, 32° 37° and 42°, and on MAGUU plates containing sucrose (1 M), NaCl (1 and 1.5 M), the DNA damaging agents DEO (0.01%) or camptothecin (5 µg/ml), the ribonucleotide reductase inhibitor Hydroxyurea (4 and 8 mM), or the microtubule poison benomyl (0.4 µg/ml). Kinase deletion mutants with similar phenotypes were retested together to allow phenotypic comparison and for figure generation. Heterokaryons generated for essential kinases were identified as described [35]. To determine the terminal phenotype of essential kinases, uninucleate spores generated from heterokaryons were inoculated in selective YG media at 32° for DAPI staining. Imaging of fixed cells was with a Nikon Eclipse E800 microscope fitted with a Perkin Elmer UltraPix FSI camera. For live cell imaging, conidia were germinated in liquid media in 35 mm glass bottom microwell dishes (MatTech) and imaged using a Perkin Elmer Ultraview ERS spinning disk confocal system configured with a Hamamatsu Orca-AG camera on a Nikon TE2000-U inverted microscope using a 60 1.40 NA Plan Apochromatic objective.

## Supporting Information

Table S1 Deletion primers and diagnostic primers.
(XLS)

Table S2 Kinase deletion strains.
(XLS)

File S1 Supplemental Figures S1–S15.
(PDF)
