# Supplementary material for: Functional Analysis of the Aspergillus nidulans Kinome
Source: PLoS One. 2013 Mar 7;8(3):e58008. doi: 10.1371/journal.pone.0058008 (PMC3591445; doi:10.1371/journal.pone.0058008)
Supplement: File S1 — Supplemental Figures S1–S15. (PDF) [file pone.0058008.s003.pdf]

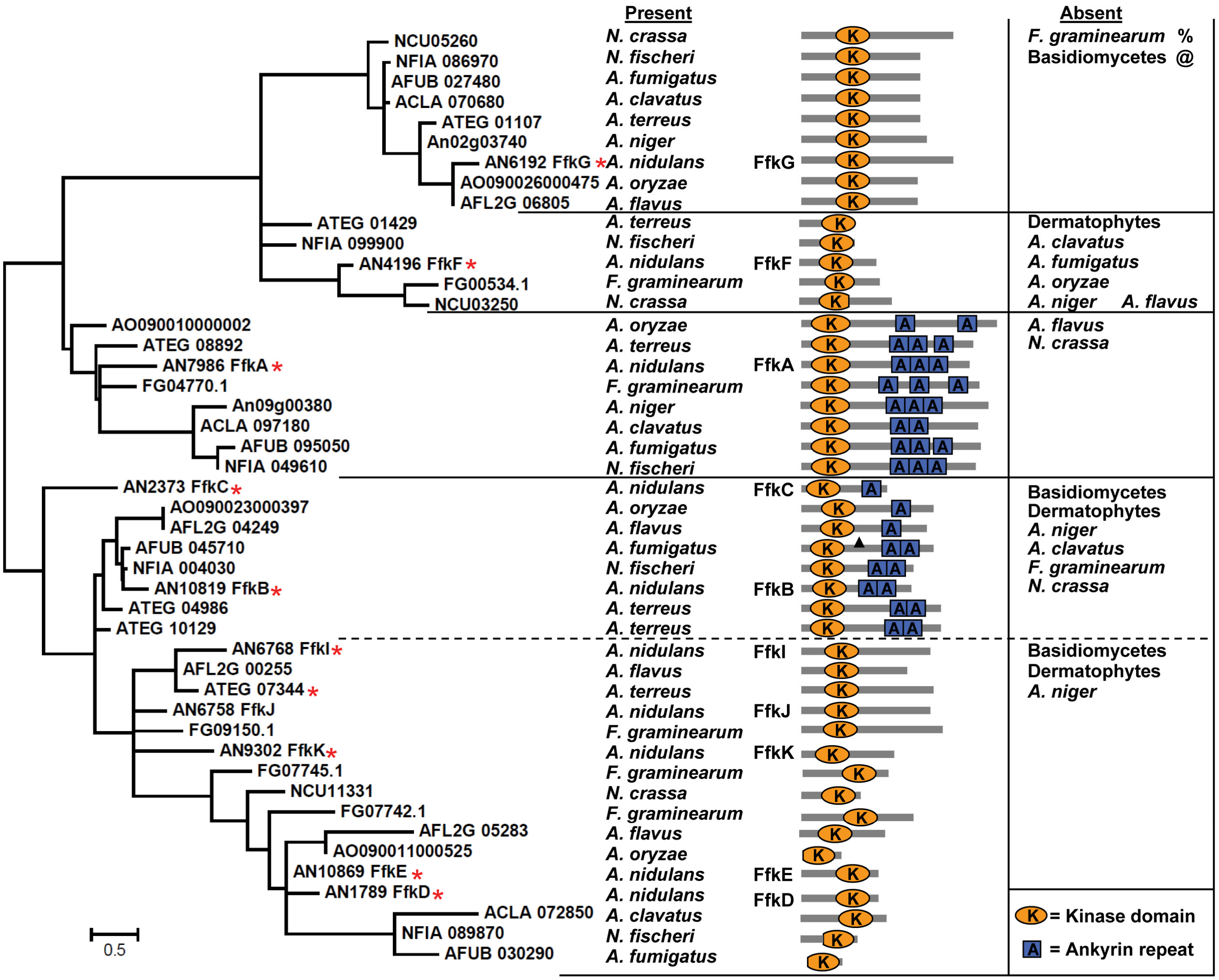

Figure S1. Rooted phylogenetic tree of Ffk kinases in the Aspergilli, *N. crassa* and *F. graminearum*. Putative orthologues were identified by reciprocal blast using the kinase domains of the *A. nidulans* Ffks (red asterisk). Kinase domains were then aligned using ClustalW (http://www.phylogeny.fr) and the tree visualized using MEGA version 5 maximum likelihood analysis. Related kinases are in groups separated by a line. Schematic diagrams show the domain organization of predicted proteins. The column on the right indicates the lack of a kinase related to the *A. nidulans* Ffks in individual organisms or in the Dermatophytes and the Basidiomycetes. % = although not identified in *F. graminearum* FfkG orthologues are present in *F. oxysporum* and *F. pseudograminearum*. @ =putative orthologues of Ffks were not identified in the basidiomycetes apart from *Laccaria bicolor* EDR04056 and EDR05258 which are related to FfkA and FfkF/G respectively. (▲)The *A. fumigatus* FfkB putative orthologue is a combination of the adjacent auto-called genes AFUB_04710 (encoding just a kinase domain) and AFUB_045700 (encodes ankyrin repeats) as the original annotation of these genes is likely incorrect. The likely pseudogene FfkH was omitted from this analysis.

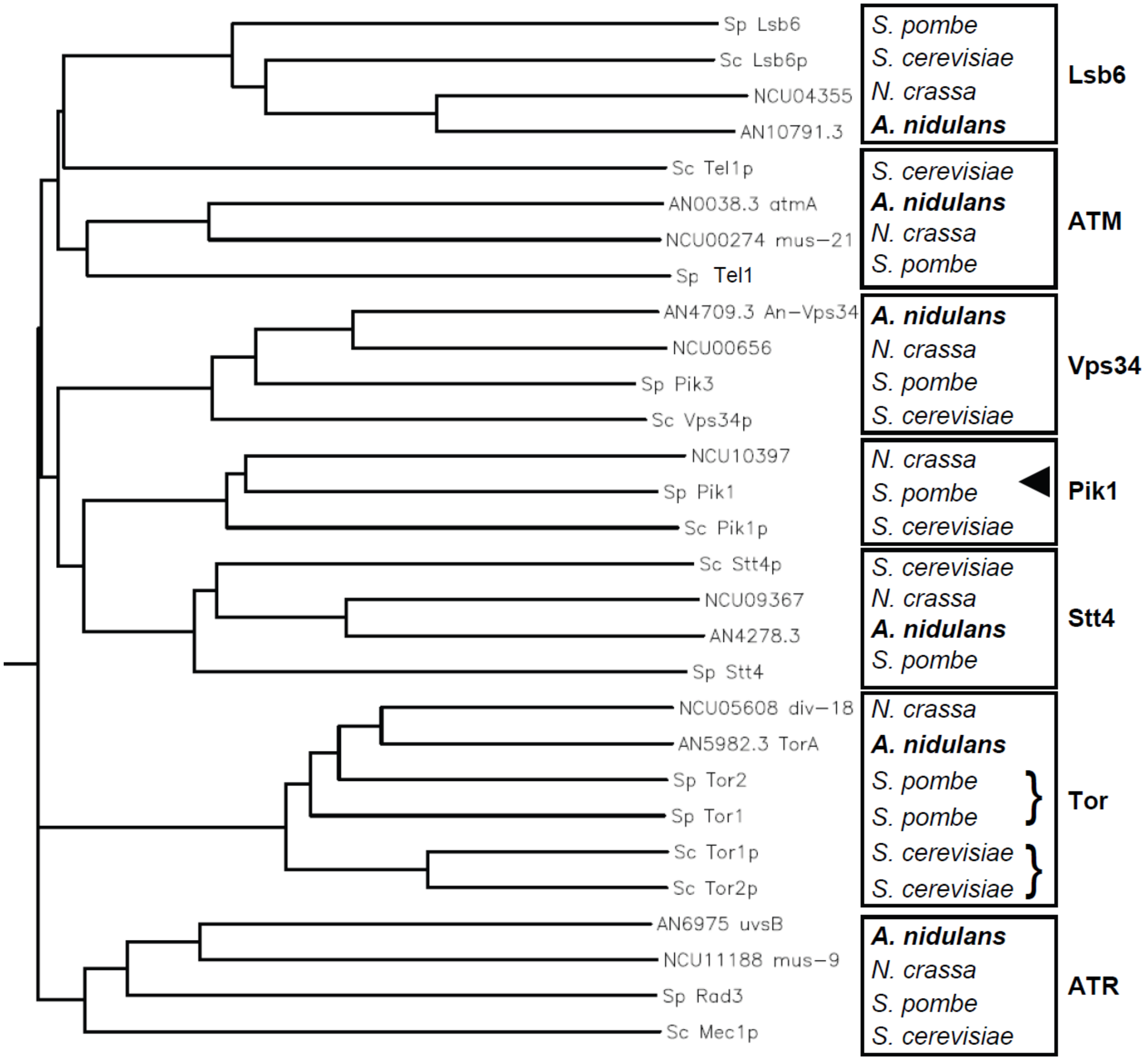

Figure S2. Rooted phylogenetic tree of the PIKK kinases from *A. nidulans*, *N. crassa*, *S. cerevisiae* and *S. pombe*. Individual PIKK kinase family members are grouped together in a box with paralogs grouped (}). (◄) The Pik1 PIKK kinase is not present in *A. nidulans* or the other 7 Aspergilli at AspGD (http://www.aspgd.org/). The tree was generated using ClustalW (http://workbench.sdsc.edu/).

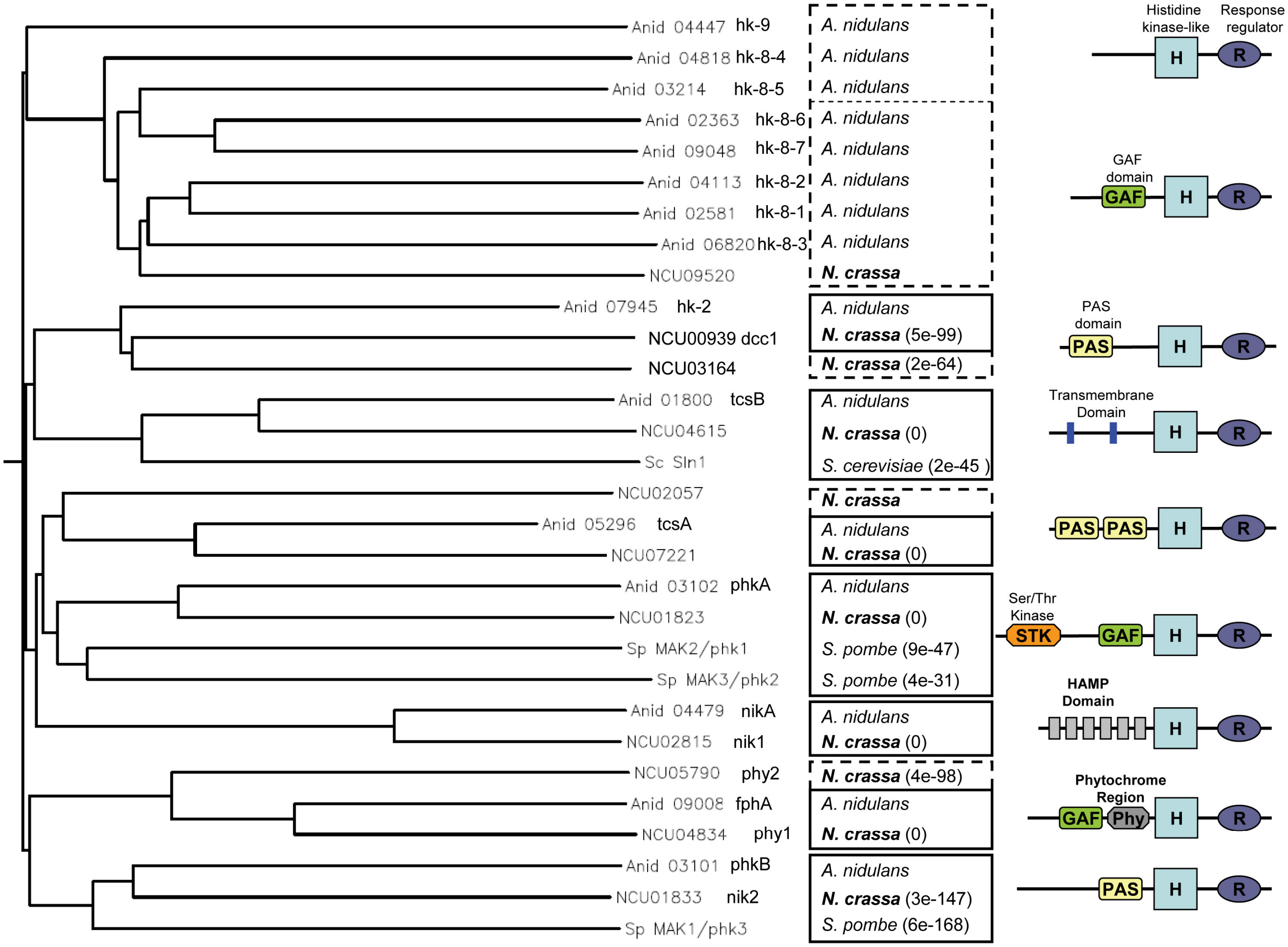

Figure S3. Rooted phylogenetic tree of histidine kinases from *A. nidulans*, *N. crassa*, *S. cerevisiae* and *S. pombe*. Predicted orthologues and/or paralogues are grouped together in a box. Boxes with broken lines indicate that the histidine kinases are likely related. Within each box the similarity of each histidine kinase to the *A. nidulans* orthologue is indicated by E-values. The domain organization of each predicted sub-family is indicated on the right [1,2]. The tree was generated using ClustalW (http://workbench.sdsc.edu/).

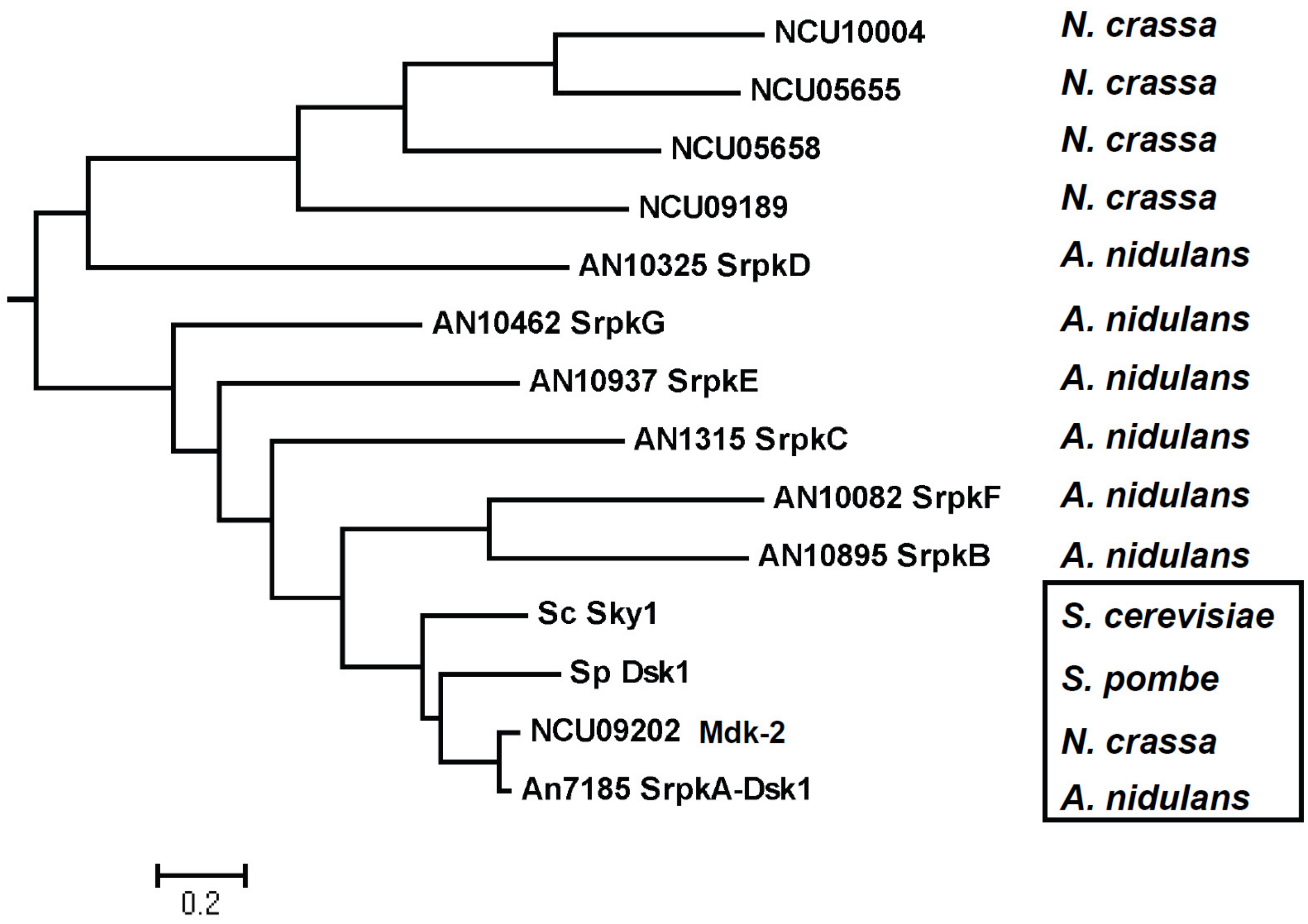

Figure S4. Rooted phylogenetic tree of SRPK related kinases in *A. nidulans*, *N. crassa*, *S. pombe* and *S. cerevisiae*. The orthologous *A. nidulans* SrpkA^{Dsk1}, *N. crassa* Mdk-2, *S. pombe* Dsk1 and *S. cerevisiae* Sky1 kinases are grouped together in a box. Alignments were generated using ClustalW (http://www.phylogeny.fr) and the tree visualized using MEGA version 5 maximum likelihood analysis.

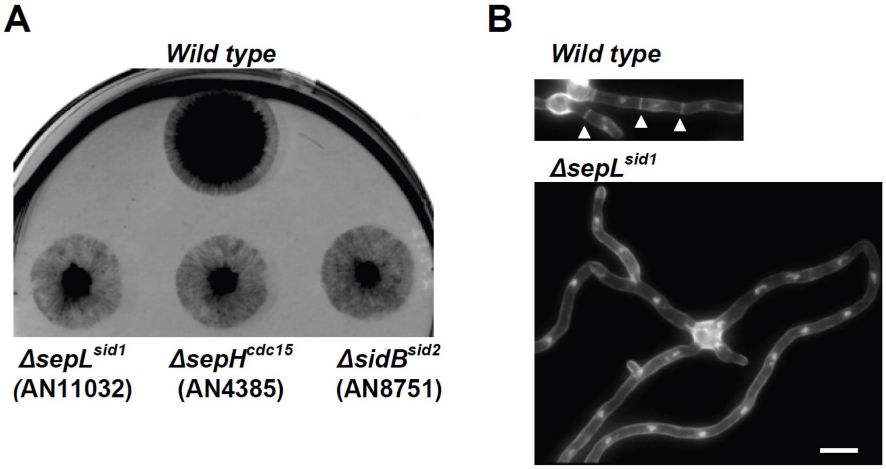

C

Genotype	Septation index (%)
Wild Type	95.6
ΔsepL^{sid1}	0.5
ΔsepH^{cdc15}	0

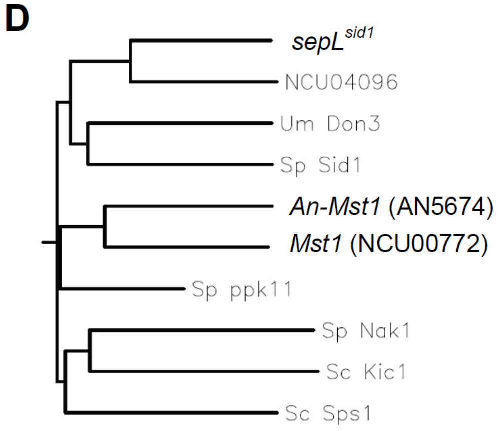

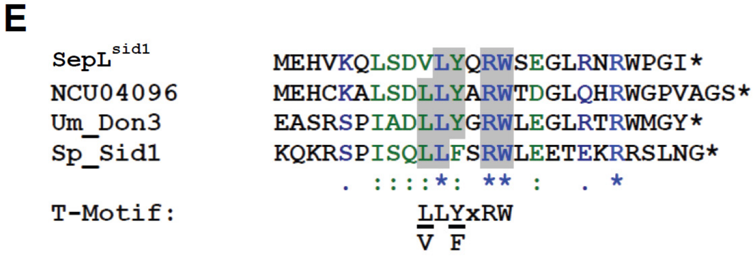

Figure S5. SepL^{Sid1} is required for septation and conidiation. (A) The predicted septation initiation network (SIN) kinase mutants display a strong growth defect characterized by extremely poor conidiation. (B) Representative images of the indicated strains grown overnight at room temperature and stained with Calcolfluor for the cell wall and DAPI for DNA. Arrowheads indicate septa present in wild type cells but not in the *sepL*^{sid1} deletion mutant. (C) Quantification of the percentage of cells with 8 or more nuclei that contained at least 1 septum (the septation index) from the same experiment as in (B). (D) Rooted phylogenetic tree showing Sid1 orthologues and related STE kinases. Shown are the *A. nidulans* kinases, their respective *N. crassa* orthologues, the *U. maydis* Sid1 orthologue Don3, and the most closely related kinases from budding and fission yeast. The tree was generated using ClustalW (http://workbench.sdsc.edu/). (E) SepL^{Sid1} contains a conserved C-terminal T-motif which is important for *U. maydis* Don3 function [3].

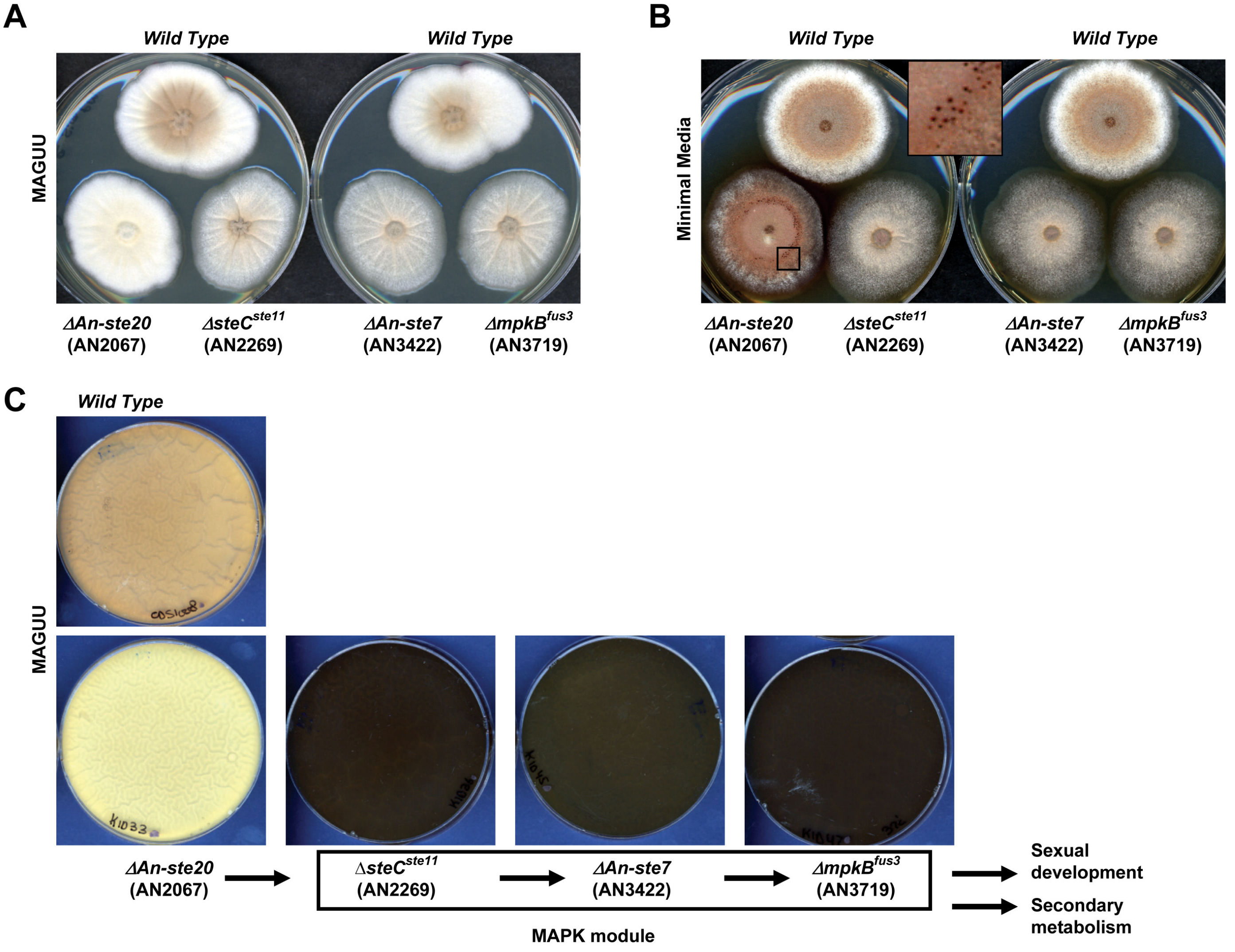

Figure S6. *An-ste20* mutants display a distinct phenotype from MAPK module kinase mutants. (A and B) Null mutations of the indicated kinases after 4 days growth at 37° on MAGUU or minimal media. The phenotype of *An-ste11*, *An-ste7* and *mpkB* mutants is identical but the *An-ste20* mutant displays a distinct phenotype. Note that on minimal media *An-Ste20* kinase mutant colonies secrete a pigment evident as aqueous droplets on the colony surface (inset). (C) MAGUU top agar cultures inoculated with 1x10^{6} spores of the indicated strains and photographed looking from underneath the plates after 6 days. The *An-ste20* mutant does not display the dark pigment produced by the three MAPK module kinase mutants. The predicted linear pathway for these mutants is shown [4,5].

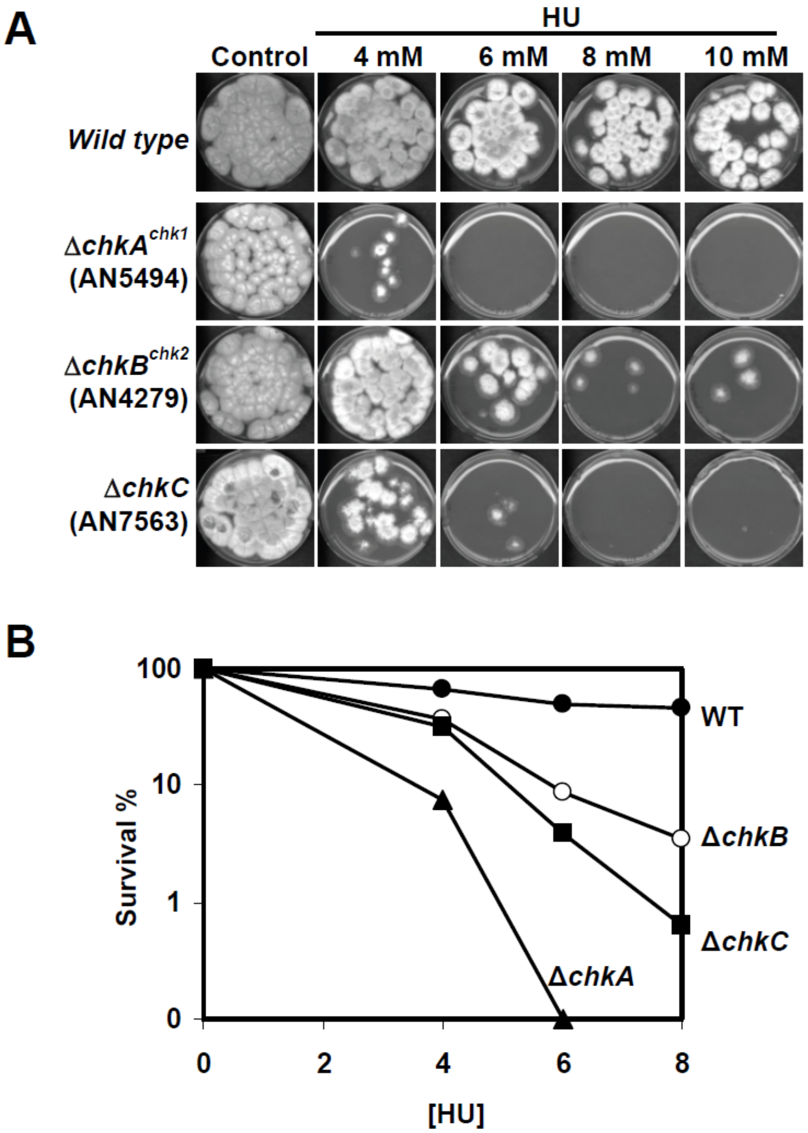

Figure S7. The 3 *A. nidulans* effector kinase mutants are sensitive to hydroxyurea (HU). (A) Plates containing the indicated concentrations of HU inoculated with an equal number of conidia from the respective strains and photographed after 3 days at 32°. (B) Graphical representation of strain viability from the experiment in A. Data points are the average of two plates at each concentration.

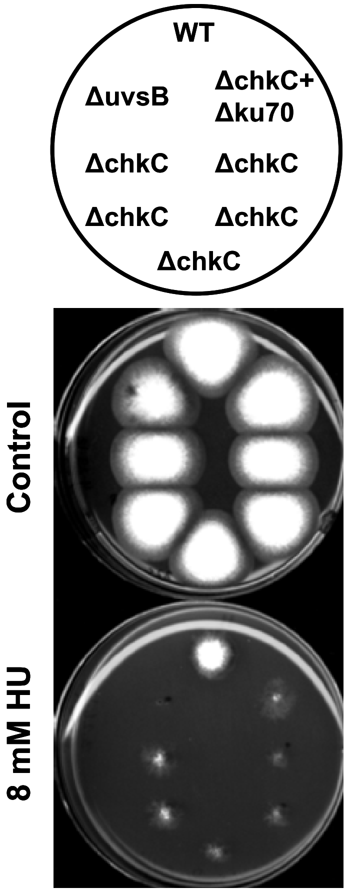

Figure S8. The HU sensitivity of *chkC* mutants is independent of the Δ*ku70* mutation. Strains with the indicated genotypes grown in the presence or absence of 8 mM HU for 3 days at 32°.

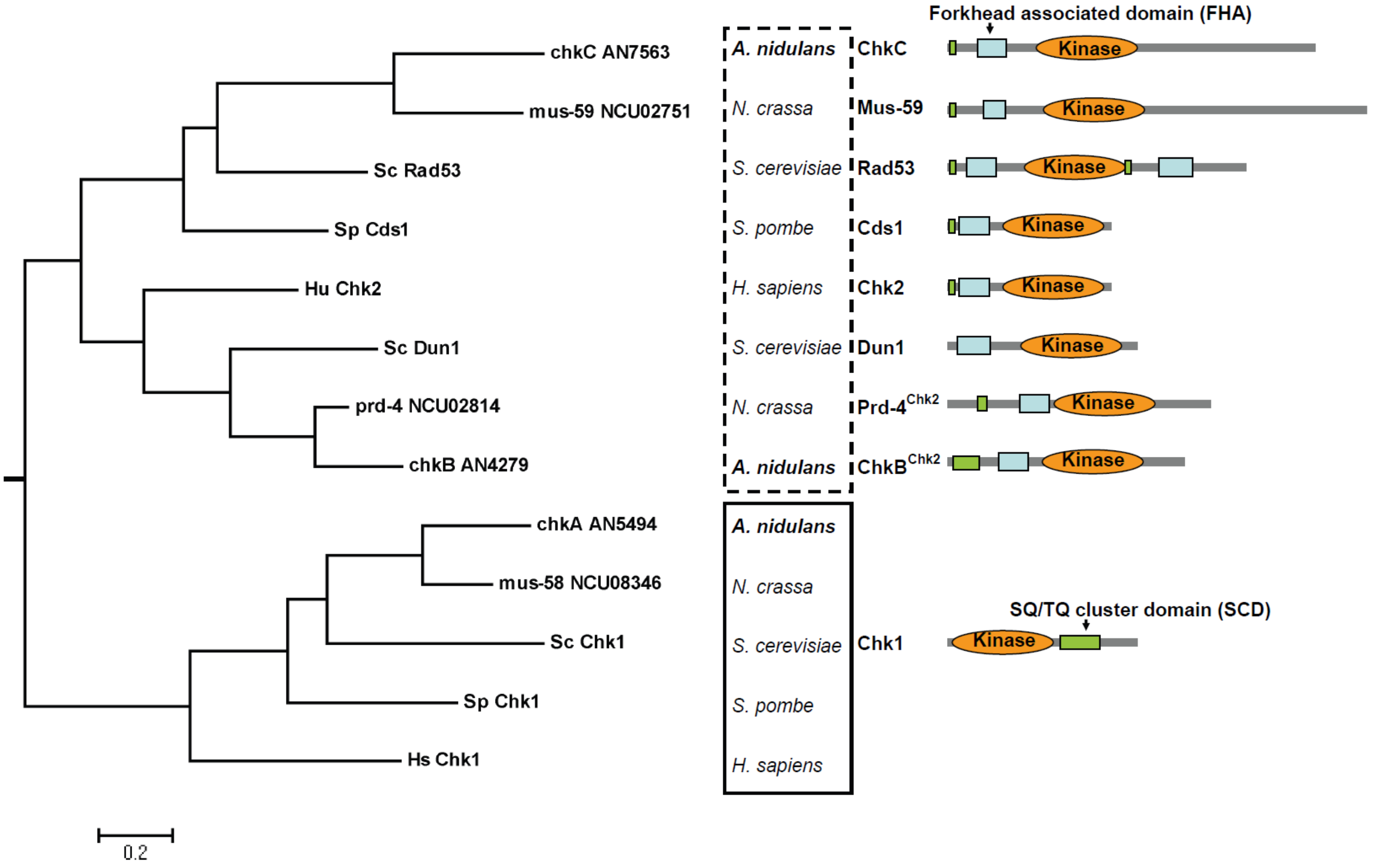

Figure S9. Rooted phylogenetic tree showing the human, *A. nidulans*, *N. crassa*, *S. cerevisiae* and *S. pombe* Chk1 and Chk2 related kinases. The tree was generated using ClustalW alignment of the respective kinase domains (http://www.phylogeny.fr/) and visualized using MEGA version 5 maximum likelihood analysis. The broken lines group together Chk2 related kinases while solid lines group together Chk1 kinases. The domain organization of each kinase is indicated on the right. SQ/TQ cluster domains (SCD) are regions containing 2 or more SQ or TQ motifs.

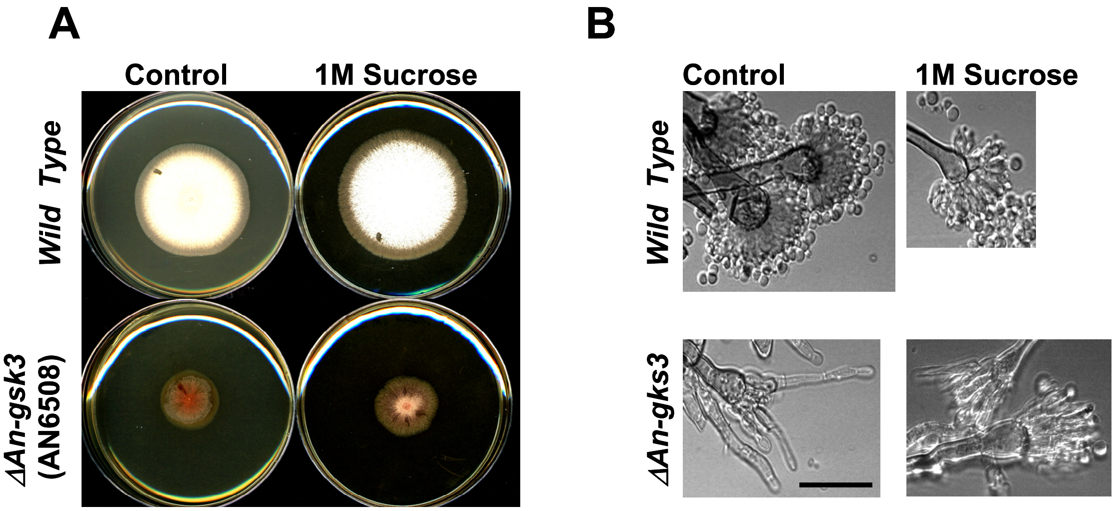

Figure S10. The poor conidiation defect of *ΔAn-gsk3* mutants is partially remediated by sucrose. (A) *ΔAn-gsk3* colonies lack the white color of the conidia but this is partially remediated in the presence of 1M sucrose. (B) Micrographs showing the highly abnormal morphology of *ΔAn-gsk3* conidiophores and the partial remediation of this phenotype by sucrose. Bar ~50 μm.

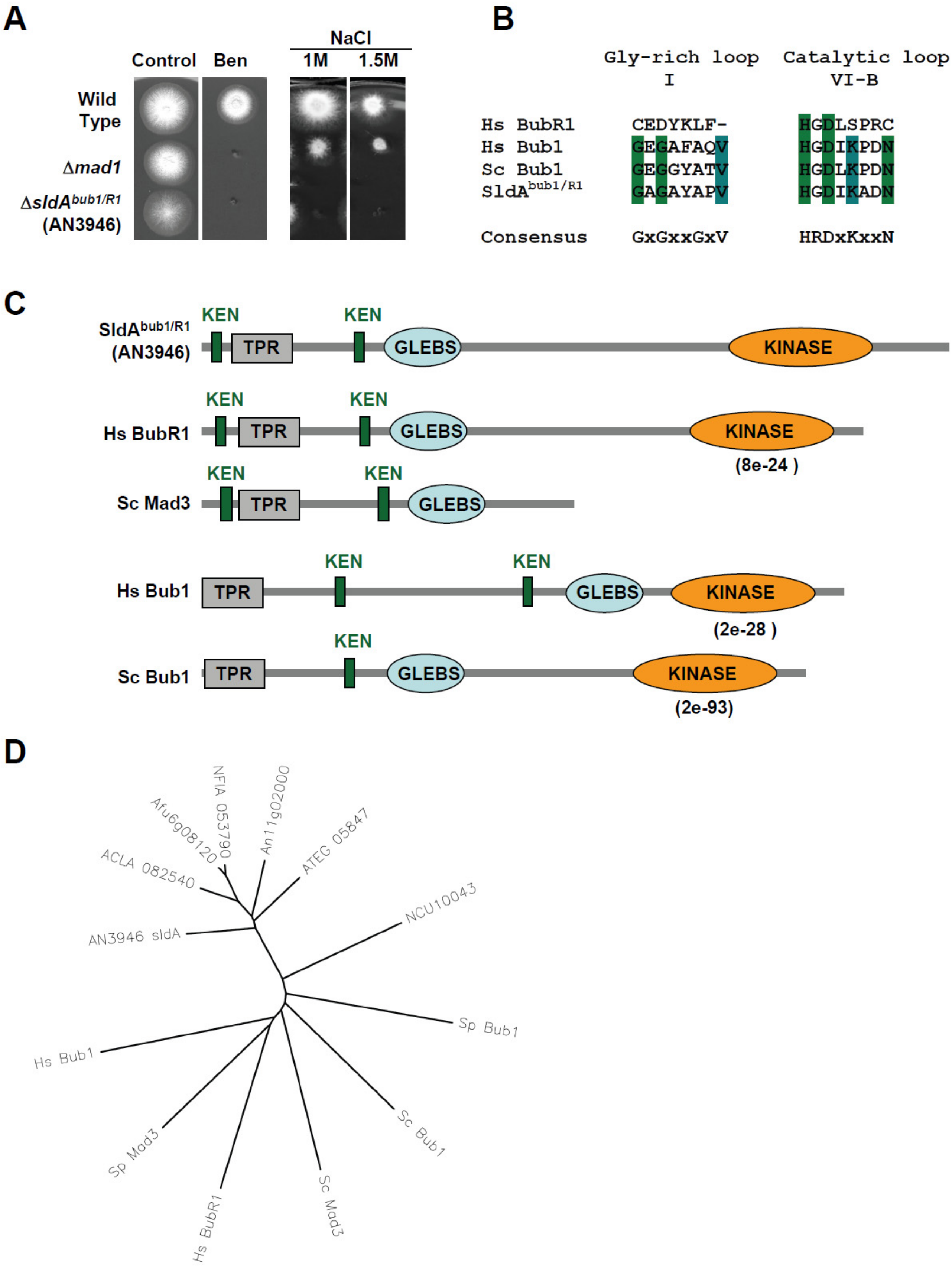
A
Control
Ben
NaCl
1M
1.5M
Wild Type
Δmad1
ΔsldA bub1/R1 (AN3946)
B
Gly-rich loop I
Catalytic loop VI-B
Hs BubR1 CEDYKLF- HGDLSPRC
Hs Bub1 GEGAFAQV HGDIKPDN
Sc Bub1 GEGGYATV HGDLKPDN
SldA bub1/R1 GAGAYAPV HGDIKADN
Consensus GxGxxGxV HRDxKxxN
C
SldA bub1/R1 (AN3946)
Hs BubR1
Sc Mad3
Hs Bub1
Sc Bub1
KEN
TPR
GLEBS
KINASE
(8e-24)
(2e-28)
(2e-93)
D
NFIA 053790
An11g02000
Afu6g08120
ATEG 05847
ACLA 082540
NCU10043
AN3946 sldA
Sp Bub1
Hs Bub1
Sc Bub1
Sp Mad3
Hs BubR1
Sc Mad3

Figure S11

Figure S11. Deletion of the spindle assembly checkpoint kinase SldA$^{Bub1/R1}$ causes sensitivity to the microtubule poison benomyl. (A) Colony formation of the indicated strains in the presence and absence of 0.4 μg/ml benomyl or NaCl. (B) Sequence alignment of the Glycine rich loop I and catalytic loop VI-B of the indicated kinase domains. Green indicates invariant residues and blue indicates highly conserved residues. The consensus is the conventional nomenclature according to the sequence of PKA. (C) Schematic diagram showing domains within SldA$^{Bub1/R1}$, human (Hs) Bub1 and BubR1, and *S. cerevisiae* Bub1 and Mad3. KEN = Ken box; GLEBS = Gle2 binding sequence; TPR = tetratrico-peptide repeat domain. The E-values are from a BLAST search of kinase domains at the Salk Institute's kinome database using SldA$^{Bub1/R1}$. Note that although the SldA$^{Bub1/R1}$ kinase domain is more closely related to Hs Bub1 than Hs BubR1, the SldA$^{Bub1/R1}$ non-catalytic domain contains several sequence features more similar to Hs BubR1. This includes the position of two KEN boxes which are important for both the function and degradation of Bub1/BubR1 proteins [6], the position of the TPR domain which is important for recruitment to kinetochores [7], and the position of the GLEBS domain which is important for the binding of Bub1/BubR1 proteins to the Bub3 spindle assembly checkpoint protein [8]. (C) Unrooted phylogenetic tree showing the relationship of the 5 proteins shown in (C) as well as SldA^{Bub1R1} orthologues from *N. crassa* (NCU10043), *A. terreus* (ATEG_05847), *A. niger* (An11g02000), *N. fischeri* (NFIA_053790), *A. fumigatus* (Afu6g08120) and *A. clavatus* (ACLA_082540). Note that while 2 related proteins are present in *S. cerevisiae* and *S. pombe* and humans, the Aspergilli and *N. crassa* encode only a single protein.

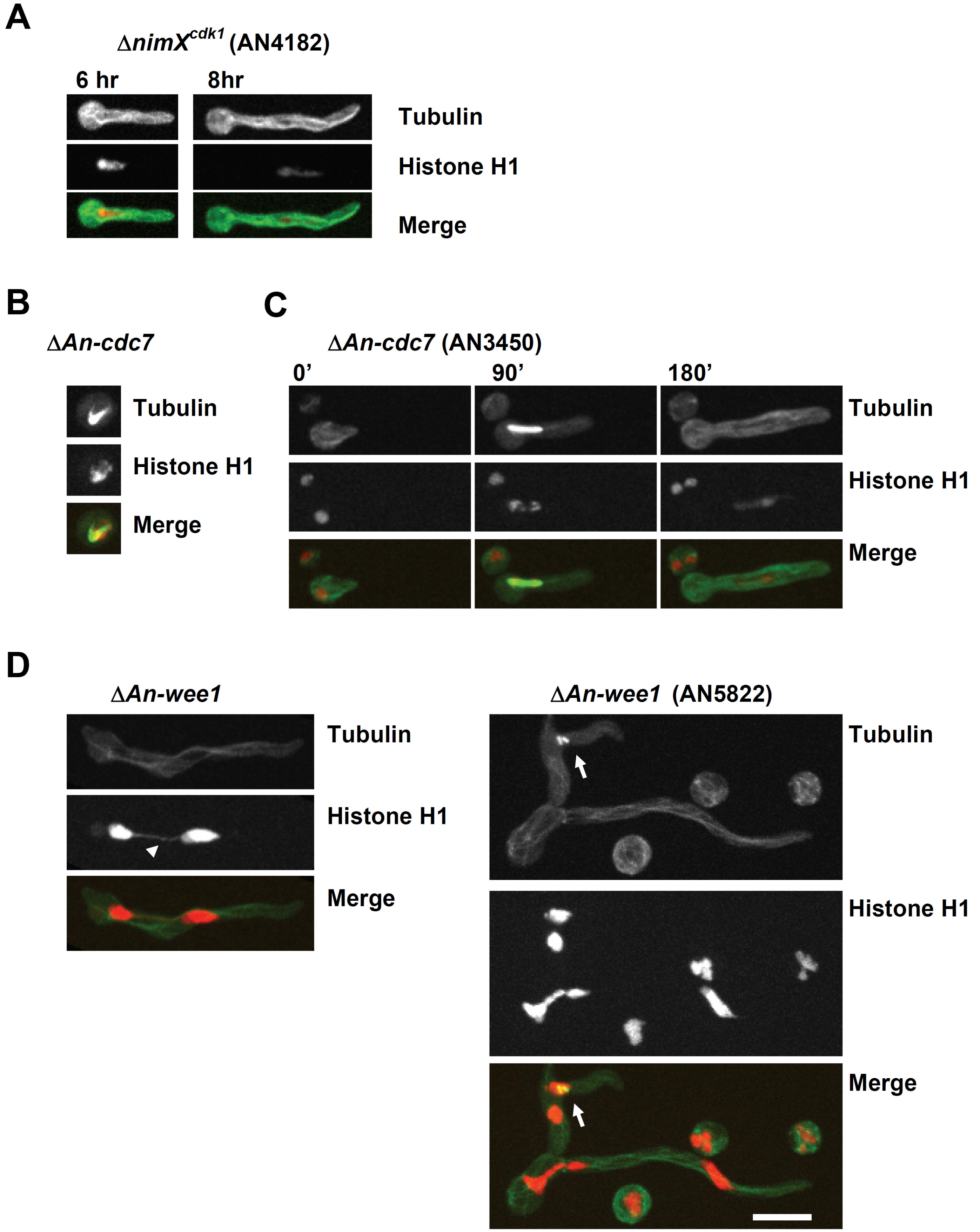
A
ΔnimX^cdk1 (AN4182)
6 hr
8hr
Tubulin
Histone H1
Merge
B
ΔAn-cdc7
Tubulin
Histone H1
Merge
C
ΔAn-cdc7 (AN3450)
0'
90'
180'
Tubulin
Histone H1
Merge
D
ΔAn-wee1
Tubulin
Histone H1
Merge
ΔAn-wee1 (AN5822)
Tubulin
Histone H1
Merge

Figure S12

Figure S12. Live cell imaging of the indicated kinase deletion mutants in a strain containing GFP-Tubulin and Histone H1-mCherry. (A) Cells lacking $NimX^{Cdk1}$ arrest with a never in mitosis (NIM) phenotype. (B and C) Cells lacking the An-Cdc7 kinase display both a NIM phenotype and a blocked in mitosis (BIM) phenotype. (B) A cell lacking An-Cdc7 displaying a monopolar spindle. (C) Time lapse imaging of a cell which enters mitosis, indicated by spindle formation and DNA condensation, but then returns to interphase without segregating its DNA. (C) Examples of cells lacking An-Wee1 which often display nuclei with an irregular size, shape and distribution. The arrowhead indicates DNA connecting two nuclei which is indicative of a failure in mitotic DNA segregation. The arrow indicates a nucleus containing two mitotic spindles. Bar ~ 15 um.

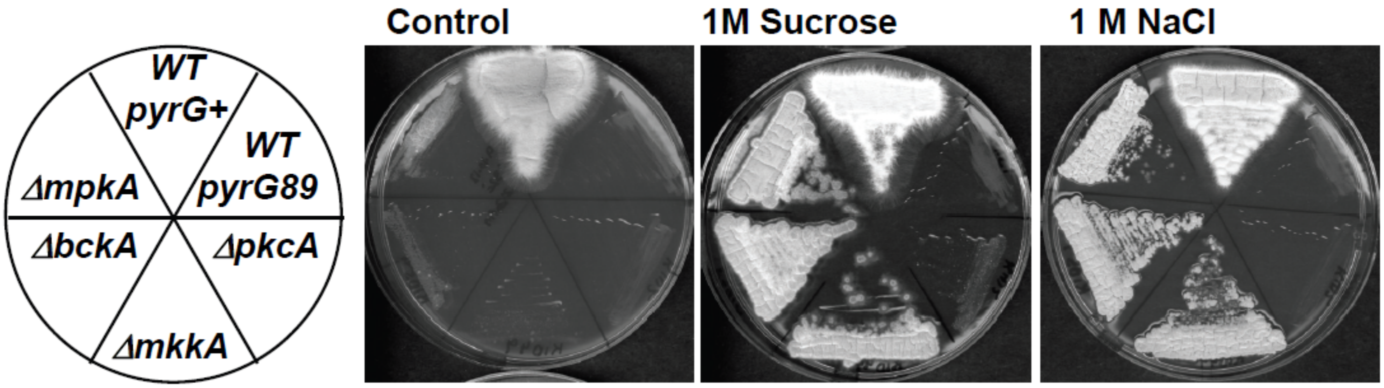

Figure S13. Rescue of cell wall integrity kinase mutants by high osmolarity. The PkcA (AN0106), BckA (AN4887), MkkA (AN4189) and MpkA (AN5666) kinases are essential and mutants do not form colonies on control YAG plates after 2 days at 32°. For the Δ*bckA*, Δ*mkkA* and Δ*mpkA* mutants this phenotype is largely remediated when osmolarity is increased by including of 1M sucrose or 1 M NaCl in the plates.

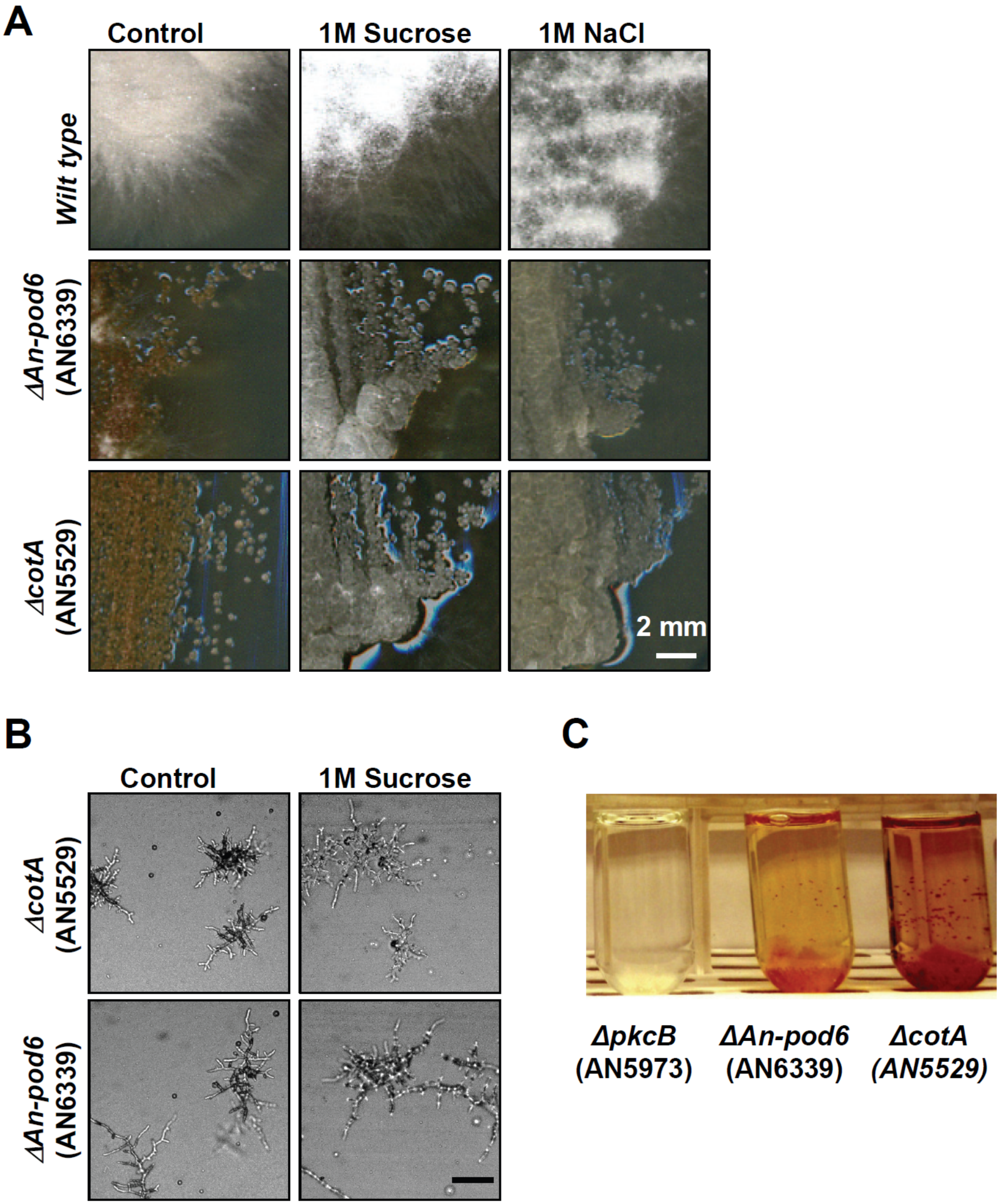
A
Control
1M Sucrose
1M NaCl
Wilt type
ΔAn-pod6 (AN6339)
ΔcotA (AN5529)
2 mm
B
Control
1M Sucrose
ΔcotA (AN5529)
ΔAn-pod6 (AN6339)
C
ΔpkcB (AN5973)
ΔAn-pod6 (AN6339)
ΔcotA (AN5529)

Figure S14

Figure S14. *An-pod6* and *cotA* nulls secrete a brown pigment. *An-pod6* and *cotA* mutants were inoculated from heterokaryons on selective media. (A) Colony formation of the indicated strains after 2 days growth at 32° with or without sucrose or NaCl. (B) Microcolonies formed by the mutants after ~ 18 hr growth with and without sucrose. Bar ~100 μm. (C) Liquid cultures of conidia inoculated from heterokaryons into minimal media selective for growth of kinase deleted cells. Cultures were grown for 4 days at 32° and photographed. Δ*pkcB* cells form microcolonies similar in size to ΔAn-*pod6* and Δ*cotA* (Figure 11) but do not secrete the brown pigment.

ΔAn-*vps15*
(AN0576)

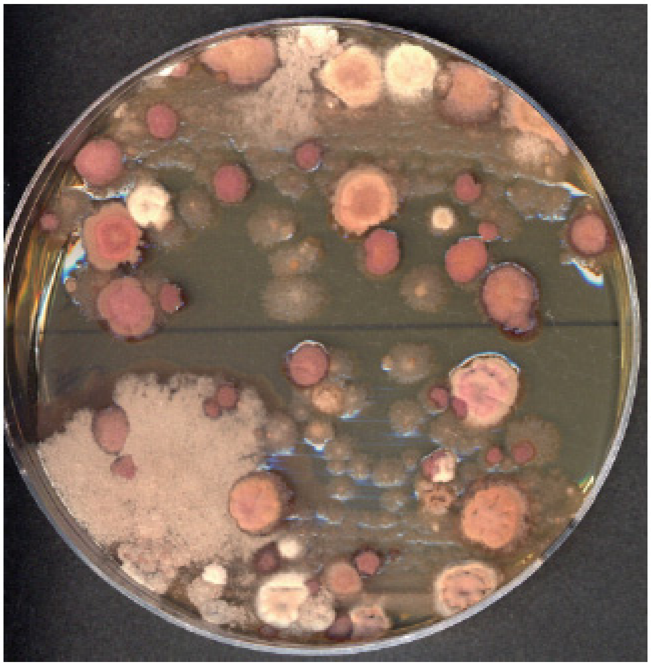

Δ*An-vps34*
(AN4709)

Figure S15. *An-vps15* and *An-vps34* mutant strains yield frequent suppressor mutations. The *An-vps15* and *An-vps34* mutants form slow growing microcolonies which occasionally show improved growth consistent with the appearance of suppressor mutations as observed for other *A. nidulans vps* mutants [9].

Primers to amplify *pyrG*^{Af} from pCDS60 for deletion constructs

**Name**	**Sequence**	**Gene**
CDS164	GATTTCAGTAACGTTAAGTGGAT	Forward primer
CDS165	GACAGAAGATGATATTGAAGGAGC	Reverse primer

Supplemental References

1. Borkovich KA, Alex LA, Yarden O, Freitag M, Turner GE, et al. (2004) Lessons from the genome sequence of *Neurospora crassa*: tracing the path from genomic blueprint to multicellular organism. Microbiol Mol Biol Rev. 68: 1-108.
2. Hagiwara D, Asano Y, Marui J, Furukawa K, Kanamaru K, et al. (2007) The SskA and SrrA response regulators are implicated in oxidative stress responses of hyphae and asexual spores in the phosphorelay signaling network of *Aspergillus nidulans*. Biosci Biotechnol Biochem 71:1003-1014.
3. Sandrock B, Bohmer C, Bolker M (2006) Dual function of the germinal centre kinase Don3 during mitosis and cytokinesis in *Ustilago maydis*. Mol Microbiol 62: 655-666.
4. Bayram Ö, Bayram ÖS, Ahmed YL, Maruyama J-I, Valerius O, et al. (2012) The *Aspergillus nidulans* MAPK module AnSte11-Ste50-Ste7-Fus3 controls development and secondary metabolism. PLoS Genetics 8: e1002816.
5. Muthuvijayan V, Marten MR (2004) In silico reconstruction of nutrient-sensing signal transduction pathways in *Aspergillus nidulans*. In Silico Biol 4: 605-631.
6. Lara-Gonzalez P, Scott MIF, Diez M, Sen O, Taylor SS (2011) BubR1 blocks substrate recruitment to the APC/C in a KEN-box-dependent manner. J Cell Sci 124: 4332-4345.
7. Krenn V, Wehenkel A, Li X, Santaguida S, Musacchio A (2012) Structural analysis reveals features of the spindle checkpoint kinase Bub1-kinetochore subunit Knl1 interaction. J Cell Biol 196: 451-67
8. Wang X, Babu JR, Harden JM, Jablonski SA, Gazi MH, et al. (2001) The Mitotic checkpoint protein hBUB3 and the mRNA export factor hRAE1 interact with GLE2p-binding sequence (GLEBS)-containing Proteins. J Biol Chem. 276: 26559-26567.
9. Calcagno-Pizarelli AM, Hervas-Aguilar A, Galindo A, Abenza JF, Penalva MA, et al. (2011) Rescue of *Aspergillus nidulans* severely debilitating null mutations in ESCRT-0, I, II and III genes by inactivation of a salt-tolerance pathway allows examination of ESCRT gene roles in pH signalling. J Cell Sci 124: 4064-4076.
